# Cellulosic Hydrogels Comprising Cellulose Nanocrystals or Chitin Nanocrystals for Historic Ceramic Conservation

**DOI:** 10.1021/acsami.6c05573

**Published:** 2026-06-12

**Authors:** Madalen Azpitarte Aretxabaleta, Marta García-Castrillo, Laura García Boullosa, Sonia Aníbarro Sánchez, Dorleta Jimenez de Aberasturi, Erlantz Lizundia

**Affiliations:** † Life Cycle Thinking Group, Department of Graphic Design and Engineering Projects, Faculty of Engineering in Bilbao, University of the Basque Country (EHU), 48013 Bilbao, Spain; ‡ BCMaterials, Basque Center for Materials, Applications and Nanostructures, UPV/EHU Science Park, 48940 Leioa, Spain; § Arkeologia Museoa, Calzada de Mallona, 2. 48006 Bilbao, Spain; ∥ CIC biomaGUNE, Basque Research and Technology Alliance (BRTA), 20014 Donostia-San Sebastián, Spain; ⊥ Ikerbasque, Basque Foundation for Science, 48009 Bilbao, Spain

**Keywords:** nanocellulose, nanochitin, renewable materials, ancient ceramics, cultural heritage, cultural conservation

## Abstract

Over time, antique ceramics undergo a series of chemical and physical degradation processes, such as salt crystallization or the accumulation of atmospheric pollutants, which compromise their structural and aesthetic integrity. To restore and conserve cultural heritage artifacts, polymeric gels have been developed, where, in comparison to conventional petroleum-derived materials, naturally occurring polymers offer enhanced properties in terms of tunable viscoelasticity, high water retention capacities, low toxicity, and inherent biodegradability. Accordingly, here, we develop nanocomposite gels based on hydroxypropyl cellulose and incorporated biocolloids with anionic and cationic surface charges, cellulose nanocrystals (CNCs), or chitin nanocrystals (ChNCs). To enhance the durability and ductility of the hydrogels, chemical cross-linking with citric acid and plasticization with ethylene glycol are performed. Tunable Young’s modulus (17.2–145.4 MPa) and elongation at break (14.3–109.1%) are obtained. Cleaning studies are carried out on actual 17th–19th century ancient ceramics from an archeological site in Bizkaia (Basque Country, Northern Spain). The hydrogels are applied directly onto the ceramics to set a continuous solid–liquid interface with no need for external mechanical pressure. X-ray fluorescence results reveal the hydrogels removing up to 72% of the lead in ceramics in 8 h of treatment, making those materials particularly suitable to remove traces of hazardous substances that are integral materials in various traditional crafts and cultural heritage artifacts. The strategy of incorporating biobased colloids within a cellulosic hydrogel allows for tuning its chemical and physical properties and introduces additional hydroxyl and amine/acetamide moieties that act as Lewis bases to enable chelation and electrostatic attraction effects for the cleaning process.

## Graphical abstract

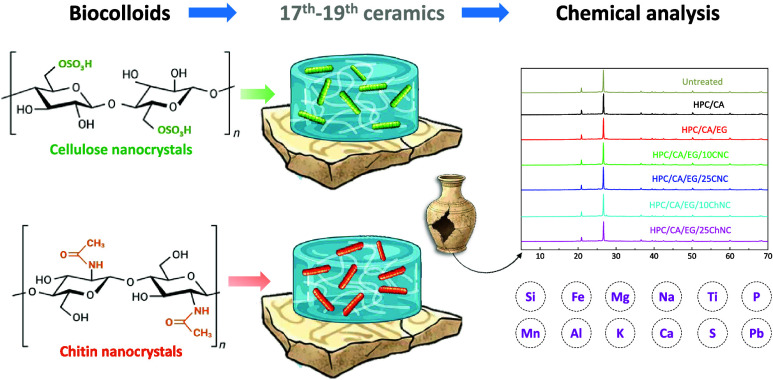

## Introduction

Over the centuries, archeological ceramics undergo a succession of chemical and physical alteration processes that gradually compromise their structural integrity.^1^ Dissolved salts (including chlorides, nitrates, sulfates, or carbonates) can migrate through the material and, after concentration, crystallize either at the surface (known as efflorescence) or within the pores and cracks (known as subflorescence).^2^ This salt crystal growth imposes mechanical stresses to the ceramic material, yielding to microfractures under repeated cycles. Furthermore, the metal ions, either from local environments or introduced through environmental contamination, can precipitate (e.g., iron oxide/hydroxide stains, copper carbonates) to damage the local microstructure or form discolored zones. Additionally, residual organic matter may release phosphates or acids (acetic, formic) that upon reaction with aluminosilicates in the ceramic further degrade the material. While preserving historical residues is essential for maintaining the archeological integrity of the ceramics (as well as to reflect its history), conservation strategies must mitigate the formation of secondary byproducts (e.g., contamination by the treatment process itself) and the prevention of adverse degradation effects (e.g., fungal growth). As a result of these combined deterioration processes that weaken their microstructural cohesion, archeological and heritage ceramics show heterogeneously altered regions, with salt-rich zones, stain zones, microcracked rims, and grain detachments.^3,4^

To addresses the physical deterioration induced by salt crystallization (e.g., subflorescence, efflorescence) or chemical deterioration (e.g., staining by metal ions), aqueous cleaning solutions have been widely explored in the conservation of cultural heritage.^5,6^ These solutions involve the deposition of wet polymeric gels, such as polyacrylamide,^7^ poly­(vinyl alcohol),^8^ or poly­(2-hydroxyethyl methacrylate)/poly­(vinylpyrrolidone),^9^ onto the surface of the cultural artifact to initiate the dissolution and extraction of the salts present. During salt extraction, the dissolved salt ions migrate back to the gel in the form of saline solutions. This transport is governed by diffusion and advection forces acting through a concentration gradient driven by Brownian motion and/or capillary water flux.^10,11^ However, as conventionally utilized polymers are petroleum-derived, concerns regarding operator safety, environmental sustainability, biodegradability, and potentially inadequate control of solvent delivery or residue formation emerge. In contrast, cellulosic gel materials present a compelling alternative thanks to their renewability, local availability, facile synthesis, and safety to operators.^12,13^ For instance, cellulosic poultices composed of relatively larger fibers (200–700 μm) have been successfully utilized for desalination purposes in cultural heritage.^10,14^ However, these materials can hardly remove the salts deep in the stratigraphy.

In this regard, there are a large number of cellulosic materials that have barely been explored in this field. Among them, hydroxypropyl cellulose (HPC), a neutral, nonionic cellulose ether, holds particular promise. HPC is water-soluble, can form gels at low solid concentrations, and is chemically benign to heritage artifacts.^15^ Its compatibility with purely aqueous systems is advantageous, particularly when working with heritage materials that are intolerant of organic solvents. Interestingly, HPC gels can deliver confined water with precise spatial and temporal control, while facilitating safe removal with minimal residue.^16^ We hypothesize that the cleaning ability of neutral polymeric gels could be upgraded upon the addition of electrostatically charged materials. Considering the “safe and sustainable by design” (SSbD) framework,^17^ nanocellulose and nanochitin emerge as versatile and sustainable alternatives to conventional synthetic cleaning agents. In particular, cellulose nanocrystals (CNCs) isolated by sulfuric acid hydrolysis of lignocellulosic biomass^18^ and chitin nanocrystals (ChNCs) isolated by hydrochloric acid hydrolysis of chitin^19^ offer high surface area, strong mechanical properties, and highly charged surfaces (negative for CNCs, positive for ChNCs), allowing their interaction with contaminants on delicate surfaces without causing physical damage. Additionally, nanocellulose and nanochitin could be processed into multiple shapes and forms (films, hydrogels, aerogels, filaments, scaffolds, 3D-printed forms, etc.), offering unpaired versatility for cultural heritage purposes.^20–24^

HPC nanocomposites containing CNCs and ChNCs can suffer from poor water resistance and mechanical brittleness unless chemically cross-linked. A plausible route is the cross-linking of the hydroxyl and ether groups of HPC and the hydroxyl groups of the nanocellulose/nanochitin via esterification with citric acid.^25^ Upon thermal activation at 130–150 °C, citric acid’s carboxyl groups react with hydroxyl groups to form ester bonds, while its third carboxyl can act as a bridging site, yielding a three-dimensional network. In comparison to synthetic cross-linkers such as isocyanates^26^ or glutaraldehyde,^27^ citric acid is nontoxic, biobased, and inexpensive. Furthermore, additives can be incorporated to face the intrinsically brittle character of HPC nanocomposites. A particularly effective strategy is the incorporation of plasticizers such as ethylene glycol,^28^ which reduce interchain hydrogen bond density and increase free volume to enable greater chain mobility under stress without catastrophic cracking. Ethylene glycol also counterbalances the brittleness introduced by rigid nanofillers by softening the matrix around stress concentration sites.

Bearing this in mind, we hypothesize that the hydroxyl and amine/acetamide groups in the nanocomposite hydrogels could establish chelating and electrostatic interactions with the ceramics, thereby removing unwanted materials from cultural heritage artifacts. Accordingly, this work introduces the use of biobased nanocomposite materials for cleaning and conserving ancient ceramics as representative cultural heritage artifacts. A neutral cellulose derivative serves as a versatile, easily processable matrix for the incorporation of either positively or negatively charged biobased colloids, yielding fully biobased gels with tunable electrostatic properties. This comparative study, not unexplored in the field of cultural heritage conservation until the date, introduces a novel class of materials for the potential cleaning of diverse artifacts, as demonstrated here on antique ceramics. Chemical cross-linking with biobased citric acid is employed to prevent undesired dissolution of the matrix during its use, while an optimized amount of plasticizer is introduced to enhance flexibility. Furthermore, we investigate the use of nanocellulose and nanochitin for the restoration of 17th–19th century heritage assets from the Archaeological Museum of Bizkaia (Spain). In particular, improved mechanical performance and enhanced interactions with hazardous pollutants, such as Pb, are achieved.

## Experimental Section

### Materials

CNCs at 8 wt % in an aqueous gel were supplied by Blue Goose Biorefineries Inc. under the name BGB Ultra CNC (Saskatoon, SK, Canada). Chitin from shrimp shells (practical grade powder) and hydroxypropyl cellulose (HPC, powder, *M*_w_ ∼100,000 g·mol^–1^) were purchased from Sigma-Aldrich. Hydrochloric acid (HCl, 37%) was obtained from Honeywell Fluka.

#### Ancient Ceramics

Actual ancient ceramics were requested to the *Arkeologia Museoa, Archaeological Museum* (Bilbao, Basque Country, Northern Spain). The list of cultural heritage artifacts is provided in Table 1. Specifically, the archeological material selected for the study originates from a site in Bizkaia (Basque Country, Northern Spain) that is significant for two reasons: the volume of material recovered and its characteristics. Specifically, the site is *Zaharra 12–14–16”* found at the municipality of Orduña, Basque Country. The interventions were preventive in nature; the remains in the subsoil were assessed by archeologists before any work that could affect them could be carried out. The *“Zaharra 12–14–16”* site is a defensive moat dating from the early Middle Ages (13th century) and was discovered in parallel to the town wall.^29^ However, the moat soon lost its original defensive purpose and was used as a rubbish dump instead. As such, this site has one of the largest volume of ceramic archeological material recovered in Bizkaia. The materials selected for this project originate from stratigraphic units dating from the 17th to 19th centuries.

**Table 1:** Cultural Heritage Ceramics Extracted from the Archaeological Site of Orduña in 2007 and Used in This Study. The Dodes of Each Piece Are Shown According to the Archaeological Site.

archaeological site	characteristics	chronology	code
ZAHARRA 12–14–16	*bowl*/*pot base [VO-LII.157.632]*	XVII–XVIII century	*A ceramic*
	*pipework [VO-LII.149.34]*	XVII–XVIII century	*B ceramic*
	*uncoated bottom part [VO.LII.157.396*	XVII–XVIII century	*C ceramic*
	*enamel honey bowl base [VO.LII.157.155]*	XVII–XVIII century	*D ceramic*
	*enamel bowl base [VO.LII.157.52]*	XVII–XVIII century	*E ceramic*

#### Chitin Nanocrystal Preparation

Chitin nanocrystals (ChNCs) were isolated from chitin powder through the acid hydrolysis method. A 300 mL solution of 3 M HCl was added to commercial chitin powder at a ratio of 1:15 (w/v), and the mixture was magnetically stirred at 85 °C for 90 min. The reaction was then quenched by the addition of 900 mL of cold water. The mixture was then subjected to three centrifugation processes (8000*g* for 10 min at 25 °C). Then, 100 mL of the solution was sonicated in a UP400 St sonicator (Hielscher) having an S24d14D sonotrode (200 W) for 8 min while being cooled on ice. The material was washed again by centrifugation. To purify the dispersion further, it was dialyzed against distilled water for a week using regenerated cellulose dialysis tubing with a molecular weight cutoff (MWCO) of 12–14 kDa (Medicell Membranes Ltd.). The ChNCs were stored at 6 °C until use.

#### Hydrogel Fabrication

HPC gels containing CNCs and ChNCs at concentrations of 0, 10, and 25 wt % relative to the HPC amount were fabricated by thermal-assisted cross-linking.^30^ First, HPC at 10 wt % was dissolved in distilled water under stirring for 60 min at 300 rpm. Then, previously dissolved citric acid was added as a cross-linker to reach a concentration of 30 wt % to the HPC content. After magnetic stirring at 300 rpm for 15 min at room temperature, 5 wt % ethylene glycol relative to the HPC amount was added as a plasticizer. The mixture was stirred for an additional 15 min, and different amounts of CNCs or ChNCs were added drop by drop. An ultrasound bath (5 min, 40 kHz) was then used to remove the air bubbles. The solutions were dried by solvent casting in 9 mm-diameter Petri dishes at 30 °C for 48 h. Finally, the films obtained were thermal cross-linking at 150 °C for 20 min to obtain water-insoluble gels. The plasticizer content, cross-linking temperature, and cross-linking time were previously optimized to fit hydrogel characteristics for final application.

#### Characterization

Fourier transform infrared (FTIR)–attenuated total reflectance (ATR) spectroscopy of the materials was performed using a Jasco FT/IR-6100 in the range of 3600–800 cm^–1^ with a resolution of 2 cm^–1^. Powder X-ray diffraction (XRD) patterns of the films were obtained in a D8 Discover diffractometer in reflection mode with Cu Kα radiation (45 kV, 40 mA). The crystallinity index (CI) of the samples was estimated according to eq 1 as

1 $$Crystallinity\,\;\,index=\frac{A_{c}}{A_{c}+A_{a}}$$

where *A*_c_ and *A*_a_ represent the crystalline area and amorphous area, respectively.

The ζ-potential of water-dispersed CNCs and ChNCs (0.02 mg·mL^–1^) at pH values of 2–12 was determined in a Malvern Zetasizer Nano-ZS. The pH was adjusted by adding 0.1 M NaOH or 0.1 M HCl. CNCs and ChNCs were observed by transmission electron microscopy (TEM) on a JEOL JEM 1400 Plus apparatus at an acceleration voltage of 100 kV. A 3 μL droplet (0.01 wt % aqueous dispersion) was deposited onto a hydrophilic EMS CF300-Cu grid (glow discharge treatment; 10 mA for 30 s in a Leica EM ACE200) and the biocolloids were negatively stained with 1% uranyl acetate for 20 s (the uranyl acetate was then removed using filter paper).

Proton nuclear magnetic resonance (^1^H NMR) spectroscopy was conducted in a Bruker AVANCE III 500 at 500.1 MHz resonance frequency. Spectra were obtained at room temperature using 20 mg, 3.6 s acquisition time, 4.5 s delay time, 7.85 μs pulse, 9014.4 Hz spectral width, and >16 scans. The molar substitution (MS) was calculated according to eq 2 as^31^

2 $$\mathrm{MS}=\frac{\int {\mathrm{CH}}_{3}}{3^{*}\int H_{1}}$$

where ∫CH_3_ and ∫H_1_ correspond to the integral areas of anomeric proton peak and methyl proton peak of hydroxypropyl, respectively. X-ray photoelectron spectroscopy (XPS) experiments were performed in a Versaprobe III Physical Electronics (ULVAC) spectrometer with a monochromatic X-ray source (Al Kα line of 1487 eV), calibrated using the 3d5/2 line of Ag at 368.26 eV. Samples were deposited and dried in air on a glass slide coated by gold. Z-alignment was performed for optimal sample height prior to each sample measurement. 200 μm spot size was used. Electron neutralization was used for charge neutralization. Elemental quantification was done on survey scan with a step energy of 0.5 eV, pass energy of 224 eV, and time per step of 50 ms. Data were analyzed by CasaXPS software (2.3.16 PR 1.6).

Field-emission scanning electron microscopy (FE-SEM) was performed on cryogenically fractured surfaces of gels using a Hitachi S-4800 instrument operating at an acceleration voltage of 5 kV. Before analysis, the samples were sputtered with a 10 nm-thin gold–palladium layer. The tensile properties of the materials were analyzed using a universal testing machine (MTC-100 from IDM) equipped with a 500 N load cell. Films measuring 15 mm in length, 5 mm in width, and 150 ± 10 μm in thickness were used, with a deformation rate of 0.5 mm·min^–1^. Average and standard deviation values were determined over five measurements.

Ultraviolet–visible (UV–Vis) spectroscopic measurements were performed using a Shimadzu UV-1280 spectrophotometer. Total transmittance experiments were conducted over a wavelength range of 200–1000 nm, with a sampling interval of 1 nm and 25 accumulations. The opacity of the films was determined from UV–Vis measurements as

3 $$\mathrm{Opacity}=\frac{A_{600}}{x\left(\mathrm{mm}\right)}$$

where *A*_600_ accounts for the absorbance at λ 600 nm and *x* is the film thickness in mm. Mean and standard deviation values are obtained from five measurements. The color parameters of the films were determined using a laboratory colorimeter 8/d (4/8 mm 4 mm, Vetus). The color coordinates L* (lightness), a* (redness–greenness), and b* (yellowness–blueness) were obtained from the reflection spectra of the samples. The total color difference (Δ*E*) was quantified according to^32^

4 $${\Delta E}^{*}=\sqrt{{\left(\Delta L^{*}\right)}^{2}+{\left(\Delta a^{*}\right)}^{2}+{\left({\Delta b}^{*}\right)}^{2}}$$

where ΔL* = (L* – L*_0_); Δa* = (a* – a*_0_): Δb* = (b* – b*_0_); and L*_0,_ a*_0 and_ b*_0_ represent the color coordinates of the controls. CIE-L*a*b* coordinates, lightness (L*), hue (h*_ab_), and chroma (C*_ab_) were obtained from the reflection spectra.^28^ Five measurements were done per formulation.

The static water contact angles of the ceramic surfaces were determined by the sessile drop method with a volume of 10 μL. The mean and standard deviation values presented correspond to five measurements. Semiquantitative analysis of the main elements in the ceramics was conducted using a wavelength dispersive X-ray fluorescence (WDXRF) instrument from PANalytical (AXIOS model) equipped with a rhodium (Rh) target. The tests were performed under a vacuum, with the powder samples being formed into a borate glass bead by melting in an induction microfurnace. The loss/gain on ignition (LOI/GOI) of the samples was calculated by subjecting an aliquot portion of each sample to 1050 °C for 60 min in a muffle furnace. For the mineralogical phase XRD analysis, the samples were analyzed by powder XRD (ground samples) on a PANalytical Xpert PRO diffractometer equipped with a copper tube (λCu_Kαmedia_ = 1.5418 Å, λCu_Kα1_ = 1.54060 Å and λCu_Kα2_ = 1.54439 Å), vertical goniometer (Bragg–Brentano geometry), programmable divergence slit, automatic sample charger, secondary monochromator, and PixCel detector. The measurement conditions were 40 kV and 40 mA. For the computer processing of the obtained diffractograms and identification of the phases present, specific PANalytical X’pert HighScore software was used in combination with the ICDD PDF2 database.

#### Statistical Analysis

Analysis of variance (ANOVA) was employed to ascertain whether any discrepancies existed in the data. A p-value of less than 0.05 was considered to be indicative of statistical significance. Tukey’s honestly significant difference test was also employed in order to analyze the data.

## Results and Discussion

### Material Development Process

Here, we develop biobased nanocomposite materials for cleaning ancient ceramic artifacts. Based on the idea that electrostatic interactions could play a relevant role in removing salt deposits (e.g., calcium carbonate appearing on stone and pottery),^33^ metal ions, and residual organic matter,^34–36^ we utilize all nonionic, negatively charged, and positively charged materials. The base material consists of a cellulose ether in which the −OH groups in the repeating glucose units have been hydroxypropylated to yield −OCH_2_CH­(OH)­CH_3_ groups. HPC is easily soluble at temperatures below its lower critical solution temperature of approximately 45 °C. Due to its high water retention rate, HPC can potentially facilitate the removal of salts and other residues. Furthermore, HPC is nontoxic and is used as a food additive (E-464). Therefore, it is well-suited to the needs of heritage conservators.^16^

For negatively and positively charged material development, as summarized in Figure 1, we take advantage of naturally occurring polymers that form fibers comprising bundles of semicrystalline regions.^37,38^ Specifically, negatively charged (cellulosic) biocolloids are obtained through the sulfuric acid hydrolysis of cellulose. This process has a dual effect. From one side, the noncrystalline regions of the cellulosic chains are preferentially depolymerized by breaking the 1,4-β-glycoside bonds to yield a mixture of soluble sugars, oligosaccharides, and low-molecular weight fragments. From the other side, anionic sulfate groups are incorporated onto the CNC surface via esterification of −OH groups by sulfate ions.^13^ Furthermore, positively charged biocolloids with free amines and −OH groups are isolated from chitin derived from crustacean shells by hydrochloric acid hydrolysis.^39^ To ensure adequate mechanical properties, such as resistance to water and ductility, the formulation incorporates a biobased cross-linker (citric acid)^36^ and a plasticizer (ethylene glycol).^40^ The resulting films are freestanding and can be easily handled, thus enabling their practical application to a diverse range of cultural artifacts. In this study, 17th–19th century ceramics, including a pipework, bowl and pot bases, a storage jar, or a pot lid exposed to fire, provided by the Archaeological Museum of Bizkaia (Spain) from its collection, are utilized.

**Figure 1:**
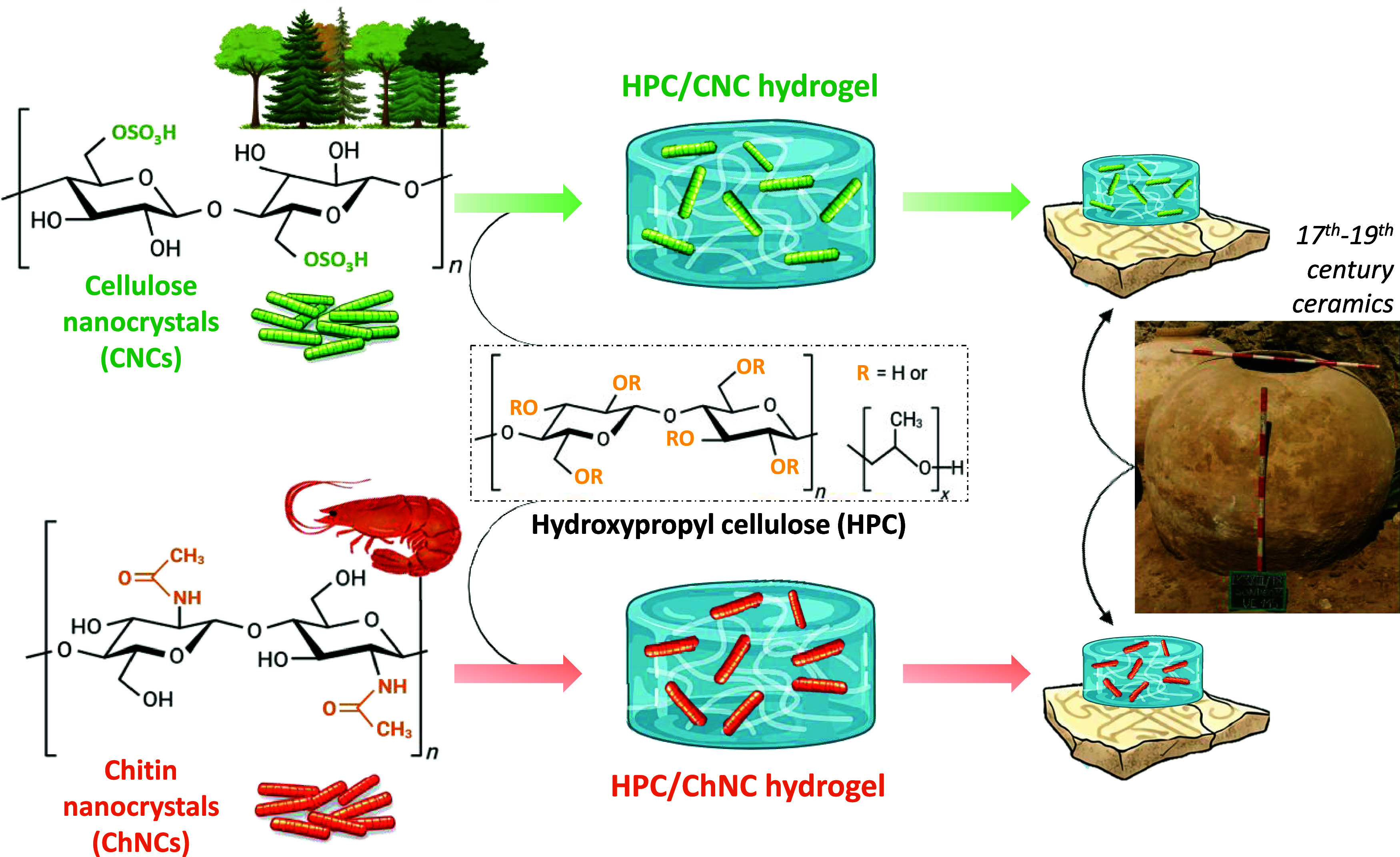
Schematic diagram showing the fabrication process of the hydrogel and its application onto 17th–19th century ceramics.

### Physicochemical Characterization of the Biocolloids

First, CNCs and ChNCs were characterized in terms of their conformational, structural, and morphological features. The attenuated total reflectance-Fourier transform infrared spectroscopy (ATR-FTIR) spectra in Figure 2a confirm the characteristic absorption bands of chitin and cellulose for ChNCs and CNCs, respectively. Both ChNCs and CNCs present a broad band at 3650–3200 cm^–1^ because of the −OH stretching. For ChNCs, the characteristic bands of the amide I, II, and II bands located at 1650, 1550, and 1310 cm^–1^, respectively, the C–O stretching at 1151 cm^–1^, and the C–N fingerprint at 895 cm^–1^ confirm the preservation of the chitin structure after the acid hydrolysis. For CNCs, the most relevant absorption bands are achieved at 1337 cm^–1^ originating from the C–O–H at C-2 or C-3, at 1160 cm^–1^ due to C–O–C bending, and at 897 cm^–1^ because of the C–O–C asymmetric stretching.^41^

**Figure 2:**
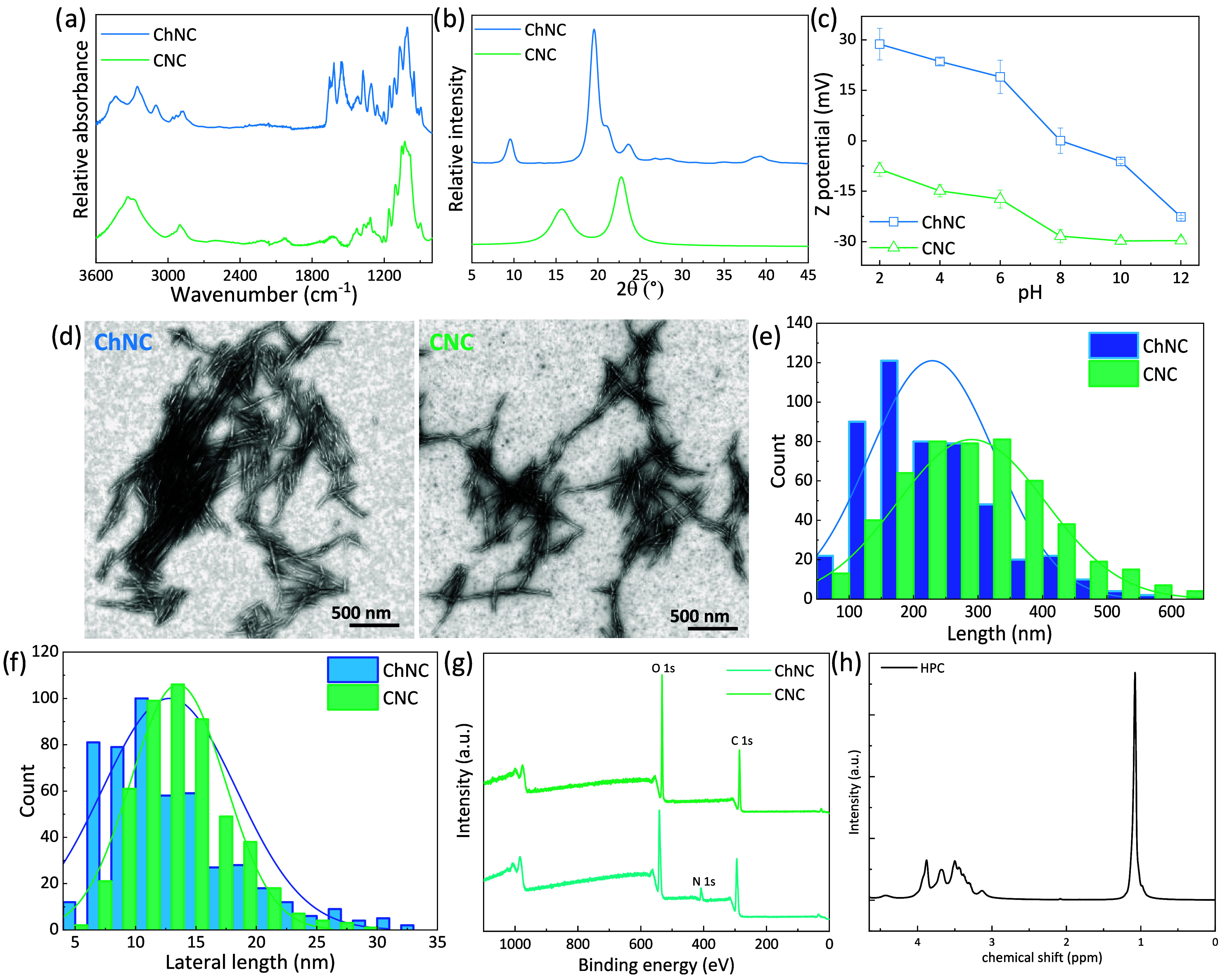
Physicochemical characterization and morphological characterization of CNCs and ChNCs: (a) FTIR spectra; (b) XRD patterns; (c) ζ-potential; (d) transmission electron microscopy (TEM) images; and (e) longitudinal and (f) lateral length distribution plot based on 500 counts from TEM micrographs. (g) Survey X-ray photoelectron spectroscopy (XPS) for CNCs and ChNCs. (h) ^1^H NMR spectra of HPC.

Both materials present a semicrystalline character, with the overlapping of a broad amorphous halo and crystalline reflections (Figure 2b). For ChNCs, main reflections occur at 2θ = 9.2 and 19.7°, which are characteristic of the (*020*) and (*110*) planes of the α-chitin form, respectively (additional weaker reflections at 2θ = 20.5 and 24.8° are seen).^42^ CNCs present solely two main diffraction peaks at 2θ = 14.9 and 22.7°, which can be identified with the (*11̅0*) and (*200*) planes of the cellulose I form, respectively.^43,44^ The crystalline index of the biocolloids suggests a more ordered structure in CNCs than ChNCs, with CI values of 80.3 and 68.9%, respectively. This is ascribed to structural differences among the polymers, where the abundance of hydroxyl groups of cellulose promotes intra- and intermolecular arrangements,^13^ resulting in a more efficient chain packaging than chitin, which shows a more complex structure containing acetamide groups.

As shown in Figure 2c, the surface charge of the two materials differs significantly. ChNCs have a higher ζ-potential than CNCs across the entire pH range studied. This is because the protonation of the *N*-acetyl groups of chitin renders ChNCs positively charged at low pH values,^19^ while the covalently attached sulfate half-ester groups make CNCs negatively charged.^18^ As suggested by these results, the colloidal stability of the two systems in water dispersions is quite different. At neutral pH, CNCs show a ζ-potential of −20 mV (thus, electrostatic repulsions will keep CNCs well dispersed). On the contrary, ChNCs are near the isoelectric point, where attractive forces dominate and aggregation effects may occur. In any case, these aggregation effects could be limited upon hydrogel formation, where the gellated HPC may physically hold the ChNCs to avoid aggregation. As demonstrated by the TEM micrographs in Figure 2d and the longitudinal and lateral length distribution plot in Figure 2e,f, both samples show a rod-like morphology. Their length is 229 ± 97 nm and 290 ± 115 nm for ChNCs and CNCs, respectively (statistics based on 500 counts), while their width remains around 12 nm for ChNCs and 13 nm for CNCs. These obtained length values match the observations for sufficiently hydrolyzed chitin from shrimp shells^19^ and CNCs isolated from microcrystalline cellulose by sulfuric acid hydrolysis.^43^

The chemical surface composition of CNCs and ChNCs was studied by using X-ray photoelectron spectroscopy (XPS), and the results are depicted in Figure 2g. Both biocolloids show two main peaks at around 533 and 286 eV corresponding to the 1s orbital electron of oxygen and carbon, respectively.^45^ A smaller peak appears for ChNCs at 409 eV, which is identified with the N 1s binding energy of acetamide groups of chitin. The relative atomic concentrations obtained from these survey spectra are 41.9% O and 59.4 C for CNCs and 34.4% O, 6.4% N, and 59.2% C for ChNCs, close to the theoretical nitrogen content of fully acetylated chitin.^46^ Figure 2h shows the proton nuclear magnetic resonance (^1^H NMR) spectrum for the commercial HPC utilized as the matrix. The results exhibited the characteristic signal of the methyl group peak of the hydroxypropyl substituents at 1.08 ppm and the cellulose backbone signals of the anomeric proton at 4.43 ppm and the −CH and −CH_2_ protons of the glucose ring between 3.13 and 3.88 ppm in ^1^H. The average number of hydroxypropyl groups attached to each glucose unit was calculated by eq 2, obtaining a value of 4.65. This value ranges in the typical range of standard and food-grade HPC.

### Optimizing the Formulation of the Biobased Nanocomposites

The development of the gels involves several materials and stages of processing with multiple variables. Therefore, an initial screening process was conducted to ascertain whether the characteristics of the resulting material are fit for purpose. First, we investigated the effect of the cross-linking time (0, 10, and 20 min) on the properties of films with a fixed EG concentration of 5 wt % and thermal treatment at 150 °C. This thermal treatment is essential to promote the esterification between −OH groups and enhance the physical stability of the material.^47^ Figure S1 shows that extending the cross-linking time results in a marked increase in Young’s modulus, although it also increases the opacity of the material. The formation of a carboxylic acid anhydride via intramolecular dehydration, which subsequently interacts with the −OH groups to form an ester group, can be observed through an increase in carbonyl ester bond absorption at 1720 cm^–1^ and a reduction in the 3300 cm^–1^ band associated with −OH groups in Figure S2.^48^ Furthermore, cross-linking the material for 20 min makes it resistant to water dissolution (Figure S3). Next, we investigated the effect of the cross-linking temperature of 130, 140, and 150 °C, using the non-cross-linked material as the control, for a fixed heat treatment time of 20 min with 30 wt % citric acid concentration. The results in Figure S4 show that increasing cross-linking temperature results in a continuous Young’s modulus enhancement at the expense of ductility, as well as a decrease in optical transparency (i.e., an opacity increase). Besides, water resistance tests in Figure S5 demonstrate the need to perform the cross-linking at 150 °C as lower temperatures fail to provide sufficient resistance to water dissolution, which could potentially compromise the safety of the cultural artifact due to contamination. Accordingly, we set a cross-linking temperature of 150 °C with a reaction time of 20 min from now on.

Furthermore, Figure S6 shows that a citric acid concentration of 30 wt % must be incorporated into the formulation, since materials with lower concentrations (5, 10, and 30 wt %) are water-soluble. Next, we investigated how the presence of ethylene glycol (0, 5, 10, and 15 wt %) affected the properties of the films cross-linked for 20 min at 150 °C. As summarized in Figure S7, EG effectively plasticizes HPC films by increasing the free volume between and promoting chain mobility,^49^ with elongation at break increasing from 46% to >300% as the EG content increases from 0 to 15 wt %. The samples also become less transparent with the presence of EG due to a mismatch in the refractive index between the composite constituents. Accordingly, an EG content of 5 wt % is considered optimum.

### Optimized Formulation

The physicomechanical characteristics of the optimized set of materials are summarized in Figure 3. The absorption FTIR bands in Figure 3a are dominated by HPC, which has a broad peak at around 3420 cm^–1^ due to −OH stretching and intra/intermolecular hydrogen bonding. Narrower bands at 2972, 1652, 1376, and 1056 cm^–1^ are also visible, which originate from the asymmetric C–O–C vibration, C=C stretching, −CH_2_ vibration, and −C–OH groups, respectively.^50^ The nanocomposite films appear similar due to the presence of common bonds. Moreover, there are no band displacements upon ChNC or CNC incorporation, indicating a lack of intermolecular interactions between the composite constituents. Besides, the nanocomposites are semicrystalline in light of the XRD patterns in Figure 3b, where amorphous and semicrystalline regions coexist. Specifically, citric acid-cross-linked HPC films present two very broad diffraction peaks over an amorphous halo due to the rupture of hydrogen bonding to enable the substitution of the hydroxypropyl moiety in the cellulose backbone.^51^ A shoulder at 2θ = 22.7° is seen upon CNC incorporation because of the (*200*) planes of the cellulose I form,^43^ while the shoulder at 2θ = 9.2° in the HCP/CA/EG/ChNC films originates from the (*020*) planes of α-chitin.^42^ A light displacement toward lower angles is seen for HPC with ChNCs, indicating an increase in the *d* interplanar spacing and that the HPC’s crystalline structure has expanded.

**Figure 3:**
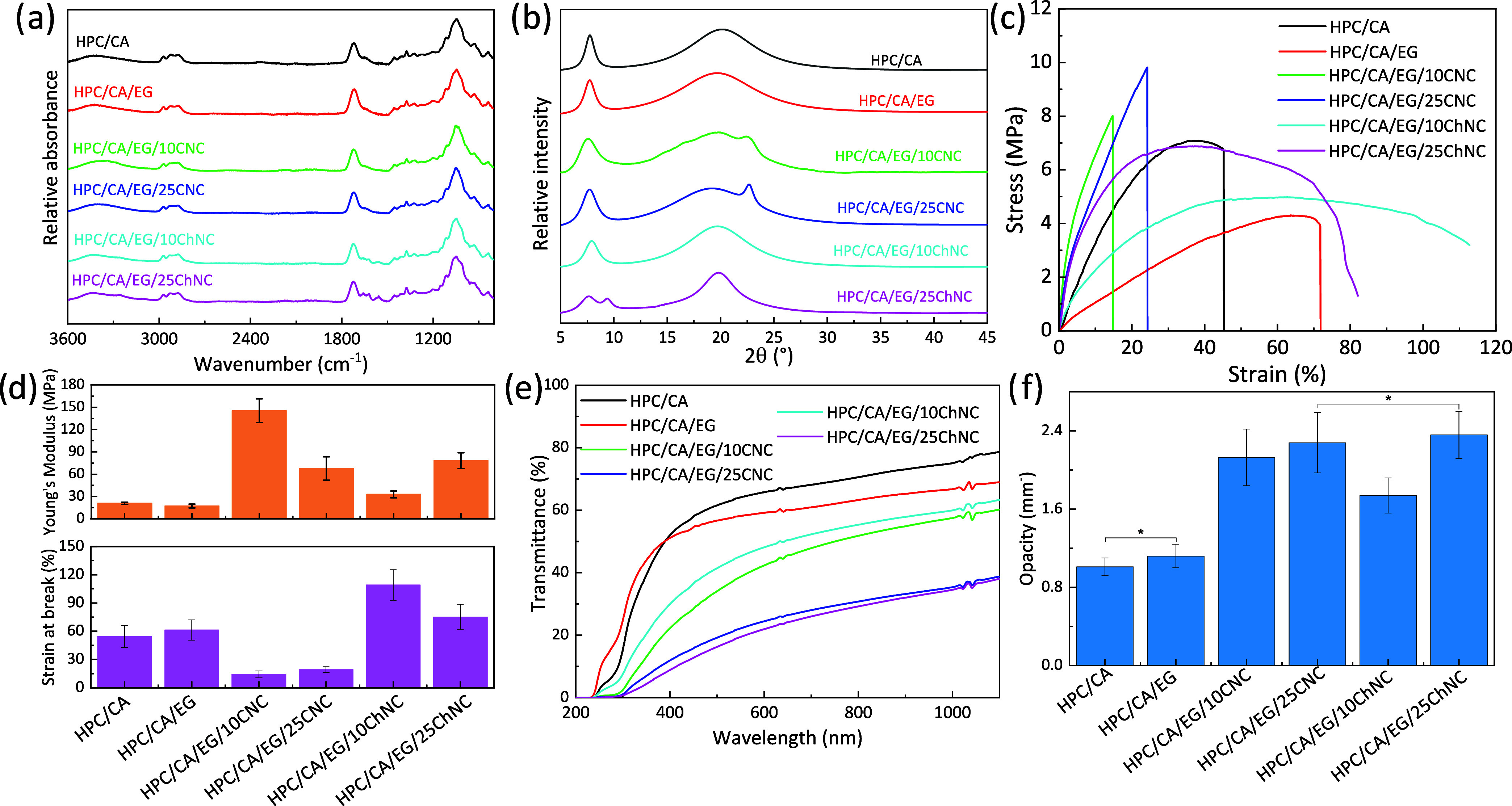
Physicochemical characterization of the HPC/CNC and HPC/ChNC nanocomposite films: (a) FTIR spectra; (b) XRD patterns; (c) uniaxial tensile testing; (d) Young’s modulus and strain at break values of HPC/CNC and HPC/ChNC nanocomposite films; (e) UV–Vis spectra; and (f) opacity values of HPC/CNC and HPC/ChNC nanocomposite films. The ANOVA test was carried out for Young’s modulus, elongation at break, and opacity values (*p-value >0.05 shows nonsignificant difference between the groups).

The morphological characteristics of the films play a key role in determining the resulting mechanical properties. Accordingly, Figures S8 and S9 show representative scanning electron microscopy (SEM) images of cryogenic cross-sections of the films. Similar surface fractures with rough features are observed for all compositions, suggesting the occurrence of ductile fracture even at high ChNC or CNC concentrations. Furthermore, the samples remained homogeneous with no aggregation of biocolloids. These results contrast sharply with those of all other biobased systems, such as those based on carboxymethyl cellulose,^49^ where micrometer-sized holes are formed throughout the film due to the high viscosity of the aqueous system. Thus, the low viscosity of aqueous HPC suspensions^52^ enables defect-free processing, representing an advantage for upscaling.

Uniaxial tensile tests were conducted to evaluate whether or not the materials developed are ductile and can withstand external forces without extensive deformation. As shown by the representative stress–strain curves in Figure 3c and the optical photographs in Figure S10, the citric acid-cross-linked HPC film is ductile and can be bent with no rupture (facilitating thus operator’s handling). This material presents Young’s modulus of 20.9 ± 1.5 MPa and a strain at break of 54.5 ± 11.7%. This behavior correlates well with literature data and is comparable to that of conventional commodity biodegradable polyesters.^51^ The addition of the EG plasticizes the material, lowering the modulus to 17.2 ± 2.7 MPa, while increasing the elongation at break up to 61.3 ± 10.8% (all the changes in modulus and elongation at break are significant in the light of ANOVA, p-value: 0.05). As expected, the addition of CNCs significantly increased the modulus to 145.4 ± 15.9 MPa for the 10 wt % formulation at the expense of ductility (the elongation at break is reduced to 14.3 ± 3.7%). Despite the usual trade-off between stiffness and toughness, ChNC incorporation simultaneously improves the modulus and ductility. We attribute this behavior to the strong interfacial and hydrogen bonding between the HPC and the functional groups of the chitin, as well as efficient energy dissipation during crack propagation provided by ChNCs (which may show localized plastic deformation).

We measured the color characteristics and opacity of the materials utilizing UV–Vis measurements because transparent materials enable the cleaning process to be observed *in situ*. Obtained UV–Vis spectra, opacity values, and colorimetric analyses are summarized in Figure 3e,f and Table S1. The opacity of the samples increases upon biocolloid incorporation due to the color changes induced by the incorporation of CNCs or ChNCs. For example, the negative ΔL* values indicate that the material has darkened relative to the reference (CIELAB color space). However, the color differences are relatively modest compared to other related systems where the incorporation of nanochitin markedly changes the color,^53^ and the opacity values are below 5 mm^–1^, which is the threshold opacity value for transparent materials.^54^ Thus, this approach offers a good trade-off between enhancing mechanical properties and maintaining optical properties.

### Cleaning of Antique Ceramics

The structural characteristics of the 17th–19th century ceramics obtained from an archeological site in Bizkaia (Basque Country, Northern Spain) were first investigated. The XRD patterns in Figure 4a and optical photographs in Figure 4b show similar features for all the studied ceramics. All of the analyzed ceramics are vitreous, predominantly amorphous, and contain crystalline phases. *A and C ceramics* display a gray color and contain rhombohedral SiO_2_ quartz (PDF file 85-798) and contain traces of phyllosilicates from the mica group (muscovite, KAl_2_Si_3_AlO_10_(OH)_2_, PDF file 7-32) and microcline (KAlSi_3_O_8_ potassium feldspar, PDF file 19-932). Additionally, the *C ceramic* also contains traces orthorhombic mullite Al_6_Si_2_O_12_ (PDF file 79-1454). From the other side, *B, D, and E ceramics* are reddish and their main crystalline phases are rhombohedral SiO_2_ quartz, rhombohedral calcite CaCO_3_ (PDF file 5-586), tetragonal gehlenite CaAl­(AlSi)­O_7_ (PDF file 74-1607), monoclinic diopside CaMgSi_2_O_6_ (PDF 75-1577), and rhombohedral hematite Fe_2_O_3_ (PDF 33-664), with traces of muscovite, microcline, and feldspar from the plagioclase group (NaAlSi_3_O_8_–CaAl_2_Si_2_O_8_, PDF file 41-1480). Besides, the *E ceramic* shows traces of a nonbasal reflection of phyllosilicates from the mica group. With regard to their elemental composition, as revealed by wavelength dispersive X-ray fluorescence (WDXRF) summarized in Figure 4c, all the ceramics are mainly composed of Si (47.0–60.7 wt %), with considerable amounts of Al (11.0–21.6 wt %) and Fe (2.3–4.8 wt %). In contrast to the gray ceramics, the red ceramics contain significant levels of Ca (5.2–15.5 wt %), with a reduced contribution of Na (0.3–0.8 wt %) and Pb (0.4–0.8 wt %). Moreover, gray ceramics have a notably higher proportion of potassium (∼3.8 wt %). On this basis, we postulate that red ceramics were produced from calcareous clays, where the coloration is achieved through an oxidizing firing atmosphere. Contrarily, the gray ceramics may have been produced from a reducing firing atmosphere with a restricted oxygen supply, where the iron is converted into magnetite that traps carbon. All the ceramics, especially the gray ones, show signs of vitrification, whereby clay particles fuse into a glass-like state. Please note that potential discrepancies between XRD and XRF results are due to the fact that XRD primarily detects crystalline phases. Therefore, it cannot provide a semiquantitative analysis of all the elements present in the ceramics.

**Figure 4:**
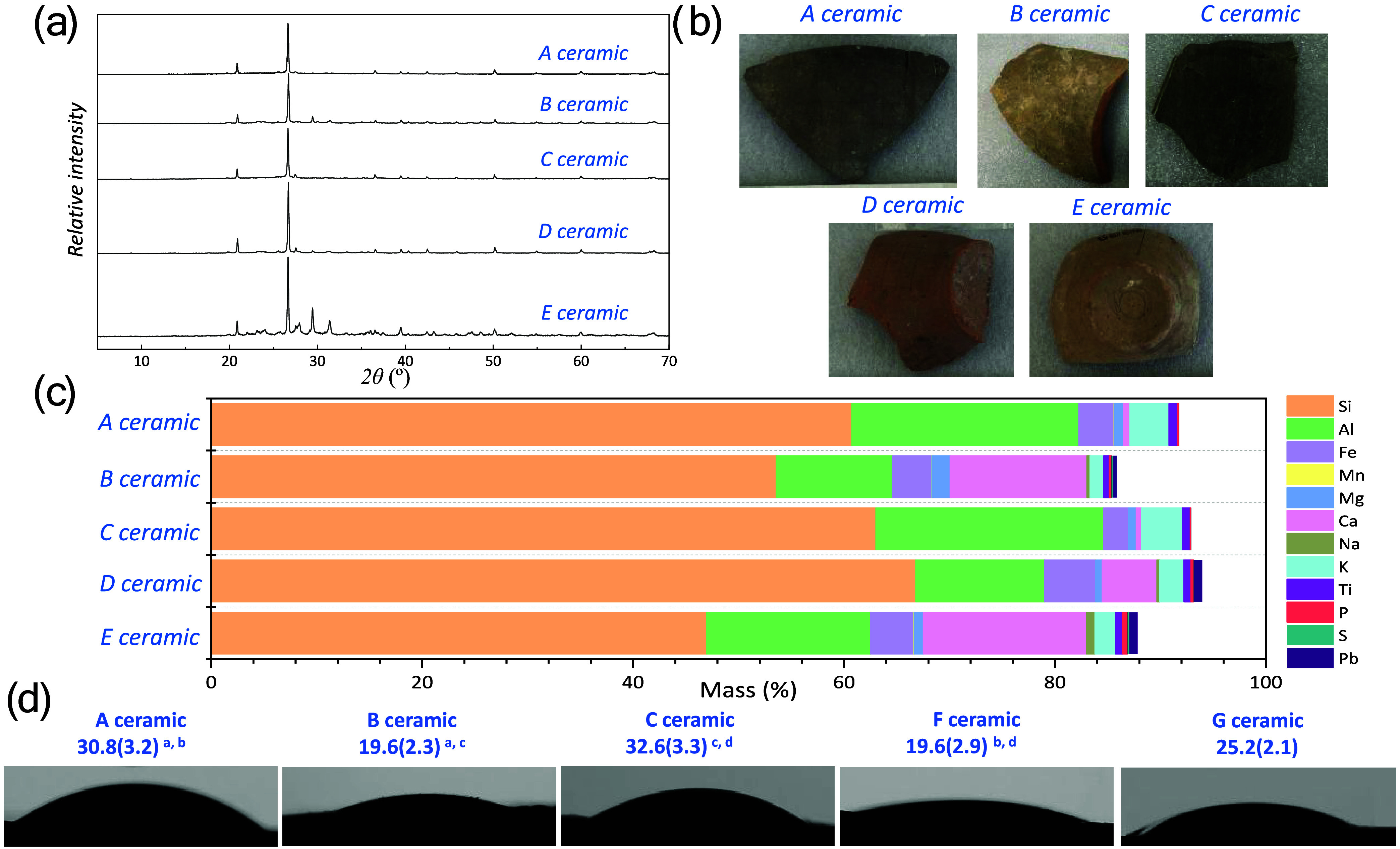
Characterization of the 17th–19th century ceramics. (a) Surface XRD patterns; (b) optical photographs of the ceramic pieces; (c) semiquantitative elemental analysis as revealed by WDXRF (the percentage that remains (up to 100%) is composed by other elements that are present in smaller amounts); and (d) optical photographs of a water droplet onto ceramics, together with their contact angle values. Mean and standard deviation values are provided; the same letter groups represent significantly different results.

Water contact angle (WCA) experiments were performed on the ceramics to determine whether relevant differences in terms of surface hydrophobicity exist among them. As seen in the optical photographs of a water droplet, Figure 4d, all the ceramics are rather hydrophilic, with WCA values varying between 19.6 and 32.6°. The reddish ceramics exhibit a higher degree of hydrophilicity, an effect that can be attributed to their calcareous composition, oxidizing firing, and clay-like characteristics, which generate polar, high-surface energy phases (e.g., calcite, gehlenite, hematite) and a lower degree of vitrification.

The ceramics were cut for convenience into small pieces of ∼10 × 15 mm, and the hydrogels were physically deposited onto the ceramics for 8 h. As shown in Figure S11, the mechanically soft character of the hydrogels facilitates an intimate interface with the ceramic being treated, setting a continuous solid–liquid interface without the need for external mechanical pressure. Note that this approach does not require utilizing the conventional barrier tissue between the cultural artifact and the treating material (e.g., Japanese paper). Upon contact, the transfer of water from the hydrogel to the ceramic is initiated by the capillary suction of the porous ceramic, and dissolution of contaminants in the ceramic is triggered so that these can migrate into the hydrogel as a solution by convective flow. The mass and color change suffered by the ceramics after the application of the gels was monitored as an indirect measure of the cleaning efficiency, and the results are summarized in Figure 5a,b in the form of heat maps. Potential moisture retention effects by the ceramics during gravimetric analyses on the calculated salt removal efficiencies were mitigated by drying the ceramic samples at 50 °C for 24 h before and after treatment. Such a gravimetric approach, where a mass loss >0.0% indicates that the ceramic experienced mass loss during treatment, is a common procedure in the field of cultural heritage, particularly to account for salt damage effects in stone artifacts, due to its simplicity but accurate and informative character.^11^ The results show that reddish ceramics undergo mass loss during treatment due to solubilization of species within the ceramic. This causes them to slowly migrate toward the interior of the cellulosic/chitinous hydrogels in the form of a saline solution. This trend could be ascribed to the higher hydrophilicity of the reddish ceramics, which could facilitate the hydrogel capability to initiate the dissolution and extraction of salts, while hydrogel performance in the gray ceramic could be inhibited by its more hydrophobic surface. Contrarily, A and C ceramics present small mass gains, probably due to the presence of small polymeric residuals on their surface once the gels have been removed.

**Figure 5:**
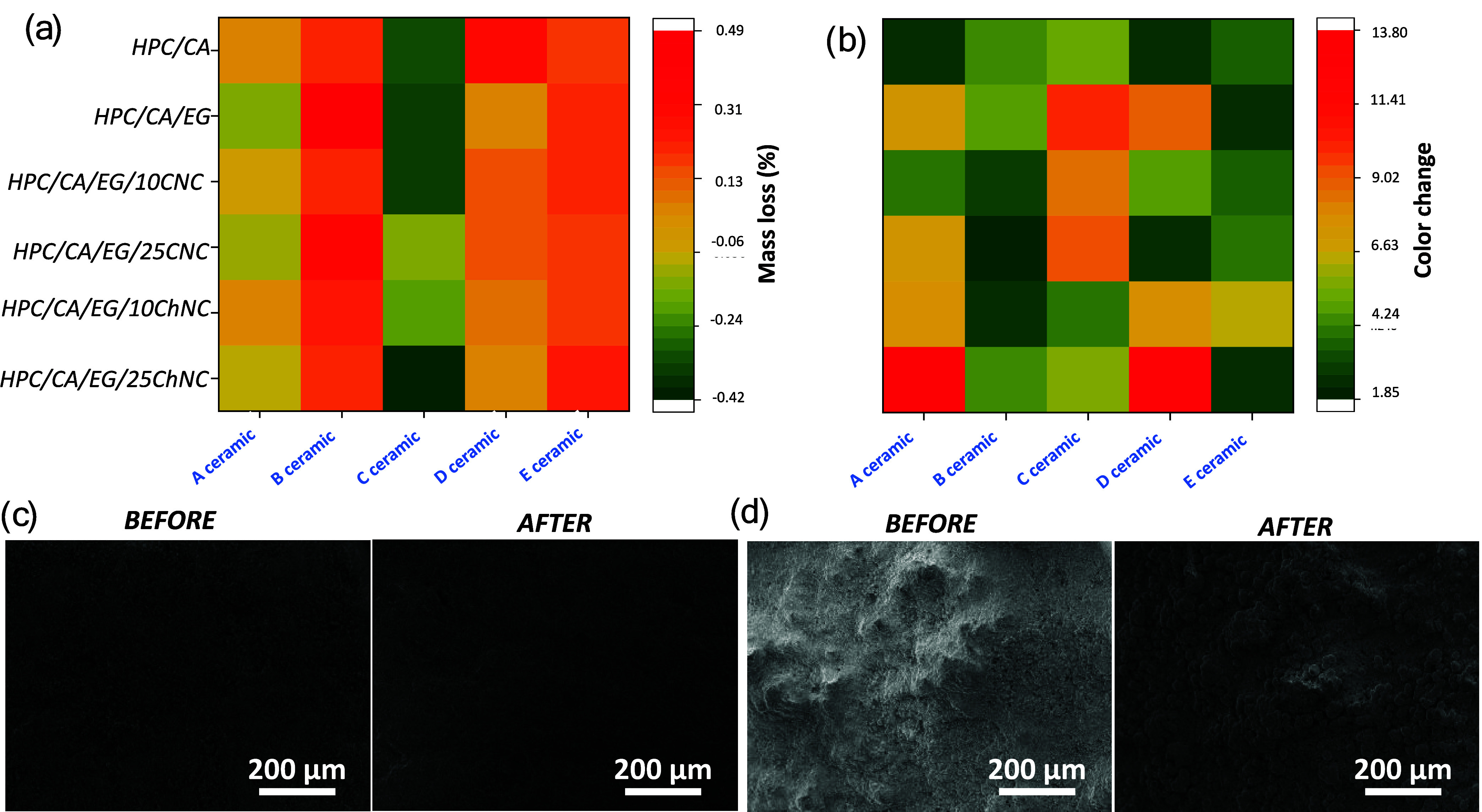
Proof of concept using real ancient ceramics from the 17th–19th centuries. Heat maps showing: (a) mass loss and (b) color change of the ceramics as a function of the treatment material. Representative SEM micrographs of HPC/CA/EG hydrogel application before and after the treatment of (c) “A gray” ceramic surfaces and (d) “B reddish” ceramic surfaces.

Color changes and SEM micrographs shown in Figure 5b–d and optical microscopy photographs of Figure S12 indicate slight morphological changes in both ceramic surfaces due to a partial adhesion of the hydrogel onto the ceramic surfaces. Thus, these observations indicate that gray ceramics (A and C) are more resistant to the cleaning (*i.e*., limited mass losses) when compared against the reddish ceramics, which show a lower degree of vitrification. When looking at the efficiency of the different hydrogel formulations according to the gravimetric analysis, CNC or ChNC incorporation does not significantly improve the cleaning capacity as the best result is obtained for the HPC/CA/EG composition. However, FE-SEM micrographs using SED (secondary electron detector) and BED (backscattered electron detector) in Figure S13 demonstrated a stronger tendency to leave hydrogel residues in materials having CNCs compared to those reinforced with ChNCs. A plausible explanation for this behavior lies in the different mechanical properties of the gels, where the enhanced ductility of the HPC/CA/EG/25ChNC formulation facilitates its deformation without rupture once the material is removed from the ceramics. On the contrary, the marked increase in the stiffness of the HPC/CA/EG/25CNC formulation makes this gel more brittle, increasing its susceptibility to fracture, thus, to leave small residues onto ceramic surfaces. Furthermore, it should be noted that very large biocolloid concentrations (e.g., 25 wt %) introduce acidic groups in the material that could alter the pH of the material being treated upon application, inducing slight color changes (Figure 5b). Therefore, these results suggest that the optimum formulation is achieved when the biocolloid (particularly nanochitin) concentration does not exceed 10 wt %.

The chemical composition and semicrystalline structure of the ceramics before and after the application of the hydrogels were further investigated through XRD and wavelength dispersive X-ray fluorescence (WDXRF) experiments, respectively. The data reveal minor changes in elemental composition (Figure 6a and Table S2) and no changes in the semicrystalline structure (Figure 6b) of the ceramics. As seen in the magnified WDXRF results in Figure S14, the semiquantitative analysis does show a small reduction in Fe (3.34 to 2.94 wt %) and Mg (0.87 to 0.82 wt %) upon hydrogel treatment for the A/C ceramics, while B/D/E ceramics show the most marked reductions in Mn (0.06 wt % below the detection point), Mg (1.72 to 1.02 wt %), P (0.18 to 0.09 wt %), and Pb (0.39 to 0.11 wt %). Conversely, the mass amounts of Na or K elements were not reduced during the cleaning process, which suggests that monovalent cations are less unlikely to diffuse from the ceramic to the hydrogels. The multivalent ion uptake may be related to a higher charge density and smaller ionic radius that facilitate the diffusion process. Despite the relatively small changes obtained, the ability to remove ∼72% of the lead present in ceramics in just 8 h of treatment is remarkable. Such preferential affinity toward lead may originate from the abundant functional groups (hydroxyls in cellulose; amine and acetamide moieties in chitin) acting as Lewis bases to facilitate chelation and electrostatic attraction. Specifically, the highly electronegative Pb^2+^ can form coordination complexes with −O and −N electron donors from the hydrogels.^55,56^ These materials may therefore be of interest for removing traces of hazardous substances such as lead, which is an integral material in various traditional crafts (e.g., stained glass, historical musical instruments). As a matter of fact, the European Chemicals Agency (ECHA) is considering adding lead to the list of substances requiring authorization under the REACH regulation.

**Figure 6:**
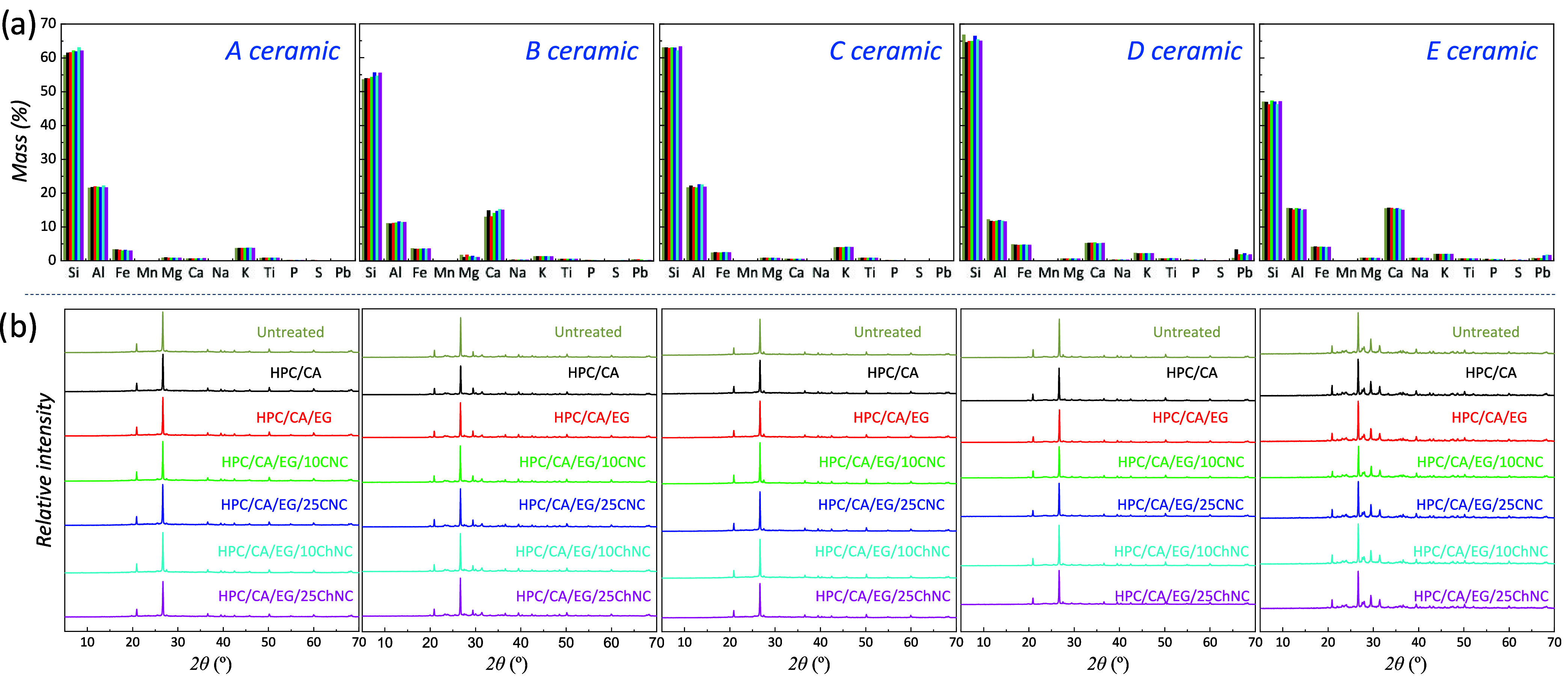
Proof of concept using real ancient ceramics from the 17th–19th centuries. (a) Semiquantitative analysis as revealed by WDXRF obtained at the surface of the ceramics. Note that for WDXRF, the values shown are relative, so an increase in a given element is due to a reduction in another. (b) XRD patterns of the same ceramics after being treated with different hydrogels.

To facilitate the practical implementation of these hydrogels, future studies should benchmark the performance with standard hydrogels such as those based on hydroxypropyl cellulose (e.g., Klucel),^57^ cellulose poultices (e.g., Arbocel),^58^ or agar.^59^ Moreover, to further boost green practices in the conservation field (see the Go Green Project),^60^ the strong mineral acids utilized during nanocellulose and nanochitin top-down isolation procedures (H_2_SO_4_ and HCl, respectively) should be replaced by milder processes that minimize chemical and energy utilization. In this sense, interesting efforts are being carried out to isolate nanochitin with no need of harsh chemical treatments (e.g., mechanochemistry)^61,62^ or to isolate nanocelluloses utilizing deep eutectic solvents,^63^ among others. These sustainable methodologies, which mitigate secondary contamination, facilitate the development of low-carbon materials, thereby aligning them with international decarbonization targets for 2030 and 2050.

## Conclusions

Here, we investigate the potential of biobased materials for the cleaning of actual 17th–19th century ancient ceramics. Hydroxypropyl cellulose is used as a model neutral water-soluble cellulosic matrix to accommodate up to 25 wt % of cellulose and chitin nanocrystals. These biocolloids were selected because of their opposite surface charges, enabling fully biobased gels with tunable electrostatic features, an area not fully explored in the field of cultural heritage cleaning. The ζ-potential of the nanochitin and nanocellulose at a pH value of 6.0 is +19.0 ± 4.9 and −17.1 ± 0.3 mV, respectively, while their average lengths are 228 and 289 nm, respectively. The addition of ethylene glycol balances the brittleness caused by citric acid cross-linking, which is crucial for making the materials water-resistant. The nanocomposite hydrogels exhibit tunable Young’s modulus values ranging from 17.2 ± 2.7 to 145.4 ± 15.9 MPa, with a tunable elongation at break ranging from 14.3 ± 3.7 to 109.1 ± 16.2%. All the materials are rather transparent as demonstrated by opacity values below 5 mm^–1^, enabling the cleaning process to be observed *in situ*. While CNC-reinforced hydrogels demonstrate an increased stiffness that enhances potential material fracture during cleaning procedures, ChNC-based nanocomposites present a synergistic improvement in both ductility and Young’s modulus. This unique combination allows the latter to deform with no rupture, ensuring an improved physical contact with the surfaces of the ceramics.

The cleaning ability of the renewable nanocomposites in short timeframes (8 h) was tested by gravimetry, colorimetry, FE-SEM imaging, X-ray diffraction, and wavelength dispersive X-ray fluorescence. In spite of the vitrified character of the treated ceramics, a semiquantitative elemental analysis reveals the removal of Fe, Mg, Mn, P, and, more importantly, Pb, from the ceramics. Importantly, ∼72% of the lead can be washed away in just 8 h of treatment, suggesting the potential of the nanocomposites containing CNCs or ChNCs for the removal of hazardous substances. The abundant hydroxyl and amine/acetamide moieties in the gels acting as Lewis bases facilitate chelation and electrostatic attraction, facilitating the cleaning process. This strategy of embedding CNCs and ChNCs to tune the physicomechanical properties of polysaccharide matrices could be extended to other cultural heritage artifacts, such as mural paintings or canvases, offering exciting possibilities for the sustainable restoration and conservation of cultural heritage.

## Supplementary Material



## Data Availability

All the data used to support the findings of this study are included within the article.

## Notes

FTIR spectra, tensile stress–strain curves, Young’s modulus values, UV–Vis transmittance spectra, opacity values, optical photographs, SEM micrographs, and WDXRF results (PDF)
