# Supplementary material for: Cellulosic Hydrogels Comprising Cellulose Nanocrystals or Chitin Nanocrystals for Historic Ceramic Conservation
Source: ACS Appl Mater Interfaces. 2026 Jun 12;18(25):35970–82. doi: 10.1021/acsami.6c05573 (PMC13339026; doi:10.1021/acsami.6c05573)
Supplement: Supplementary file 1 [file am6c05573_si_001.pdf]

Supporting Information

Cellulosic Hydrogels Comprising Cellulose Nanocrystal or Chitin Nanocrystals for Historic Ceramic Conservation

Madalen Azpitarte Aretxabaleta,[1] *Marta García-Castrillo,*[2] *Laura García Boullosa,*[3] *Sonia Aníbarro Sánchez,*[3] *Dorleta Jimenez de Aberasturi,*[4,5] *Erlantz Lizundia* [1,*]

[1] *Life Cycle Thinking Group, Department of Graphic Design and Engineering Projects, Faculty of Engineering in Bilbao. University of the Basque Country (EHU), Bilbao 48013, Spain.*

[2] *BCMaterials, Basque Center for Materials, Applications and Nanostructures, UPV/EHU Science Park, 48940 Leioa, Spain.*

[3] *Arkeologia Museoa, Calzada de Mallona, 2. 48006 Bilbao, Spain.*

[4] *CIC biomaGUNE, Basque Research and Technology Alliance (BRTA), 20014 Donostia-San Sebastián, Spain.*

[5] *Ikerbasque, Basque Foundation for Science, 48009 Bilbao, Spain.*

* Corresponding author: erlantz.liizundia@ehu.eus

Number of pages: 9

Number of tables: 2

Number of figures: 14

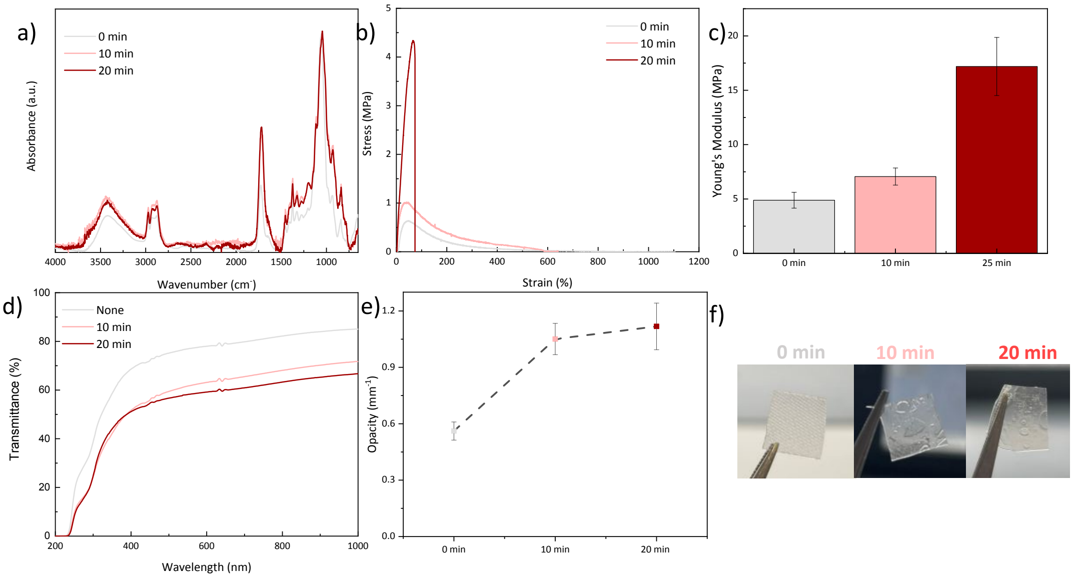

Figure S1. Cross-linking time as the variable: a) FTIR spectra; b) tensile stress-strain curves; c) Young´s modulus values; d) UV-Vis transmittance spectra; e) opacity values; and f) optical photographs.

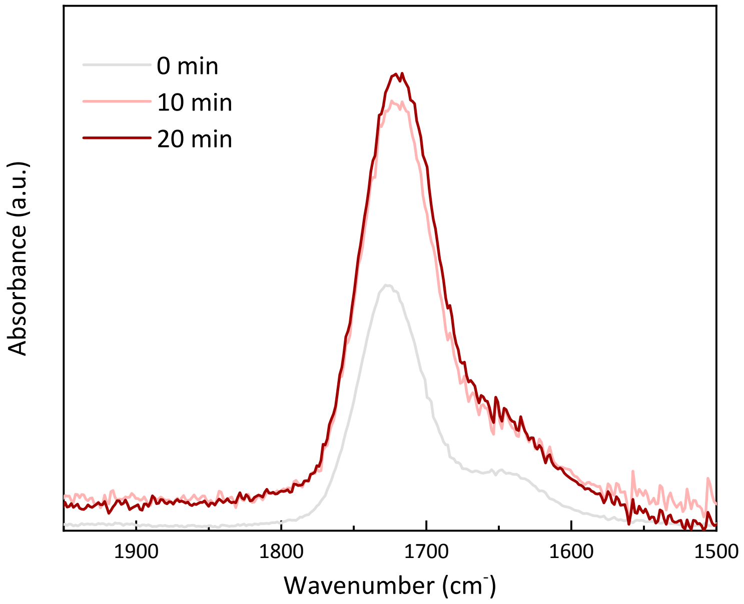

Figure S2. Magnified view of the FTIR spectra for HPC films cross-linked at different times.

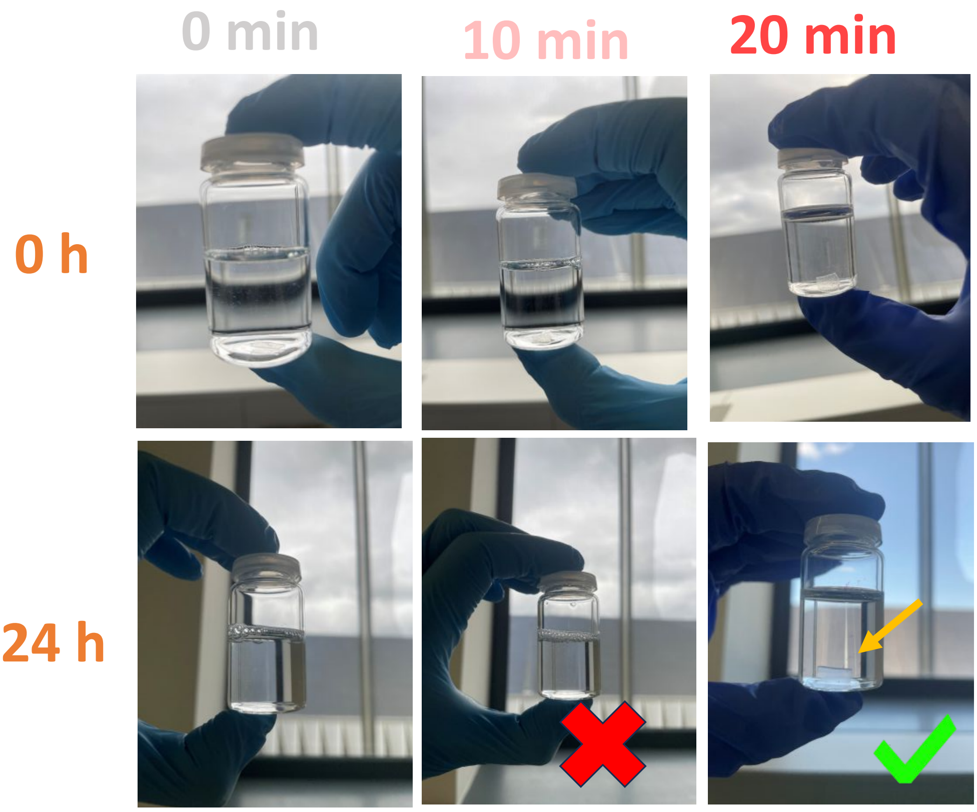

Figure S3. Optical photographs of HPC films upon immersion in water at time 0 and after 24 hours. Cross-linking time as the variable.

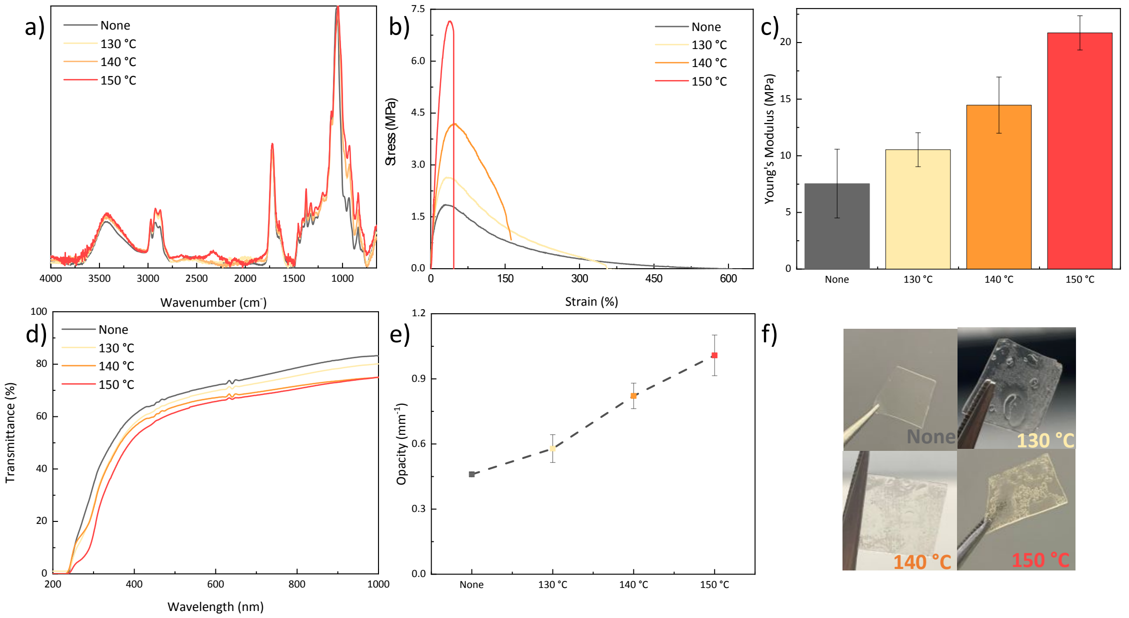

Figure S4. Cross-linking temperature of HPC films as the variable: a) FTIR spectra; b) tensile stress-strain curves; c) Young´s modulus values; d) UV-Vis transmittance spectra; e) opacity values; and f) optical photographs.

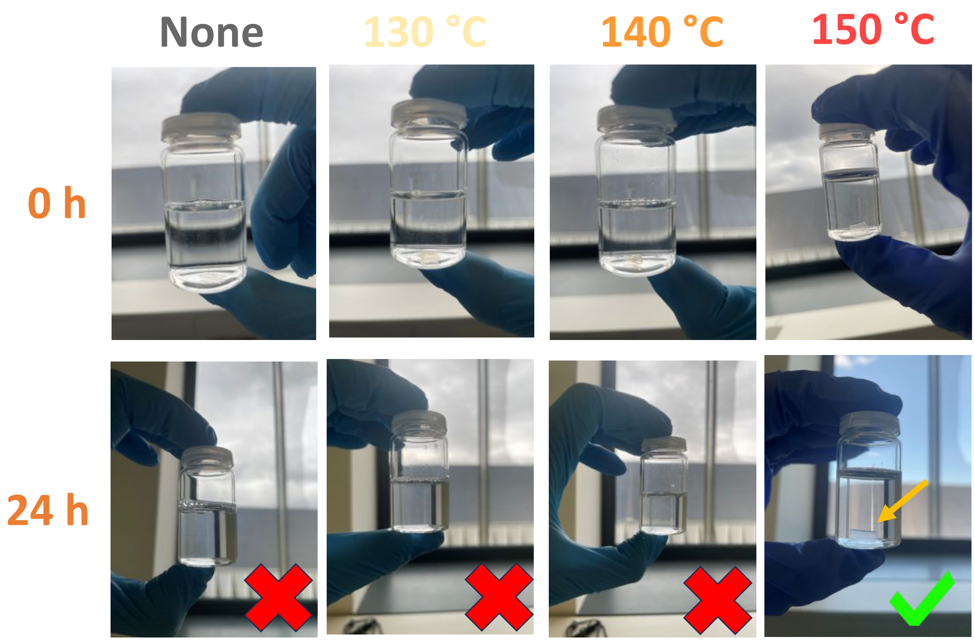

Figure S5. Optical photographs of HPC films upon immersion in water at time 0 and after 24 hours. Cross-linking temperature of HPC films as the variable.

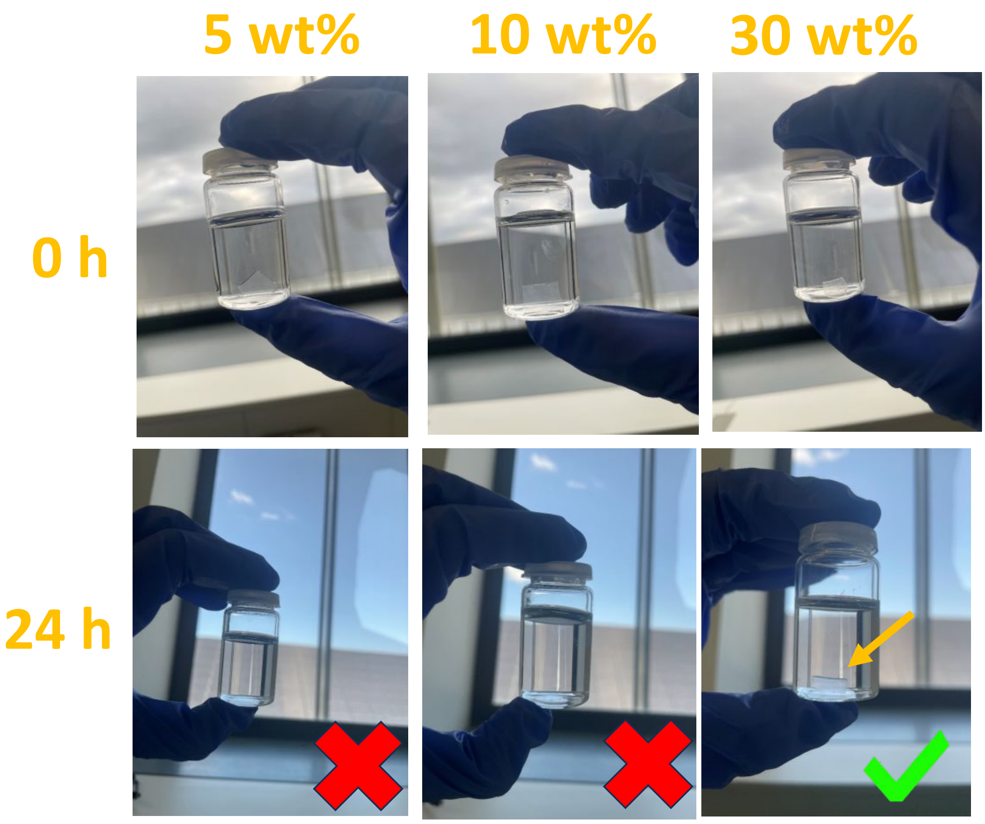

Figure S6. Optical photographs of HPC films upon immersion in water at time 0 and after 24 hours. Citric acid concentration as the variable.

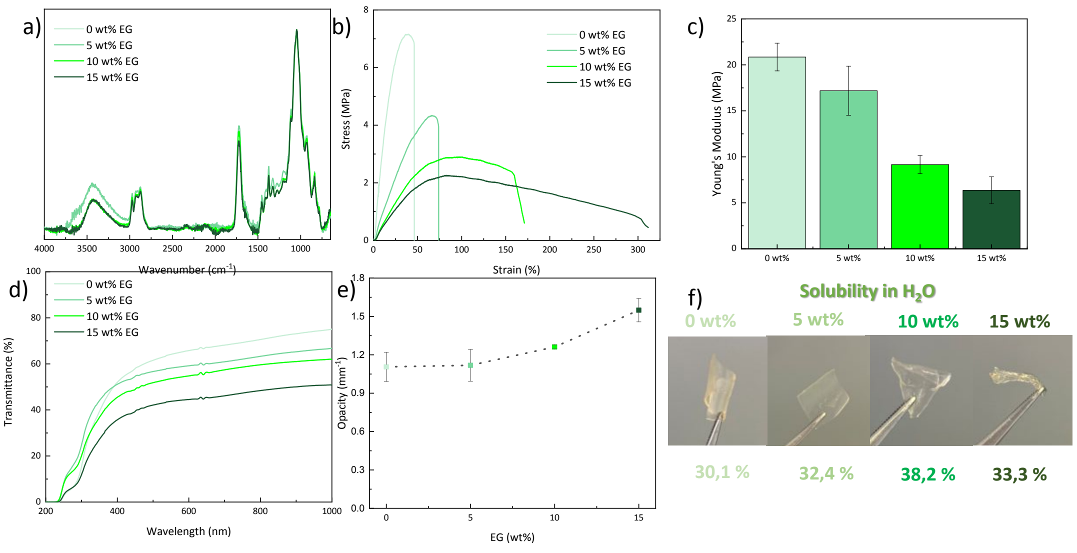

Figure S7. Ethylene glycol (EG) concentration as the variable: a) FTIR spectra; b) tensile stress-strain curves; c) Young´s modulus values; d) UV-Vis transmittance spectra; e) opacity values; and f) optical photographs.

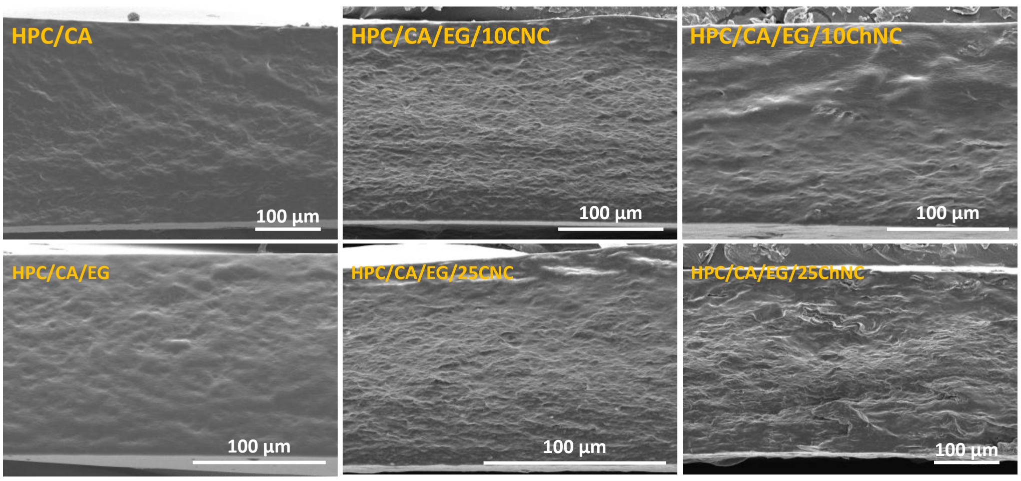

Figure S8. Representative SEM micrographs showing the morphology of the cross-section for the cryo-fractured films.

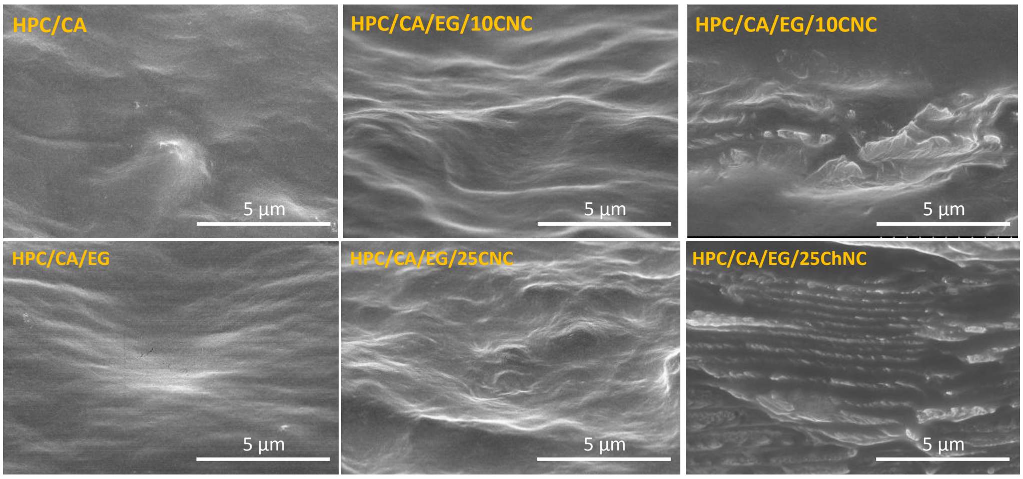

Figure S9. High-magnification representative SEM micrographs showing the morphology of the cross-section for the cryo-fractured films.

Table S1. Color properties of the films. Lightness difference (ΔL*), chroma difference (ΔC*), and color difference (ΔE*), are reported relative to the HPC/CA film.

	**ΔL***	**ΔC***	**ΔE***
**HPC/CA**	0,00 ± 0,00	0,00 ± 0,00	0,00 ± 0,00
**HPC/CA/EG**	3,24 ± 0,44	- 3,41 ± 0,35	4,73 ± 0,03
**HPC/CA/EG/10CNC**	- 1,45 ± 0,66	5,25 ± 0,71	5,46 ± 0,84
**HPC/CA/EG/25CNC**	- 0,51 ± 0,40	3,90 ± 0,53	3,97 ± 0,56
**HPC/CA/EG/10ChNC**	0,88 ± 0,63	2,63 ± 0,83	2,90 ± 0,59
**HPC/CA/EG/25ChNC**	- 3,21 ± 0,50	10,06 ± 0,79	10,57 ± 0,88

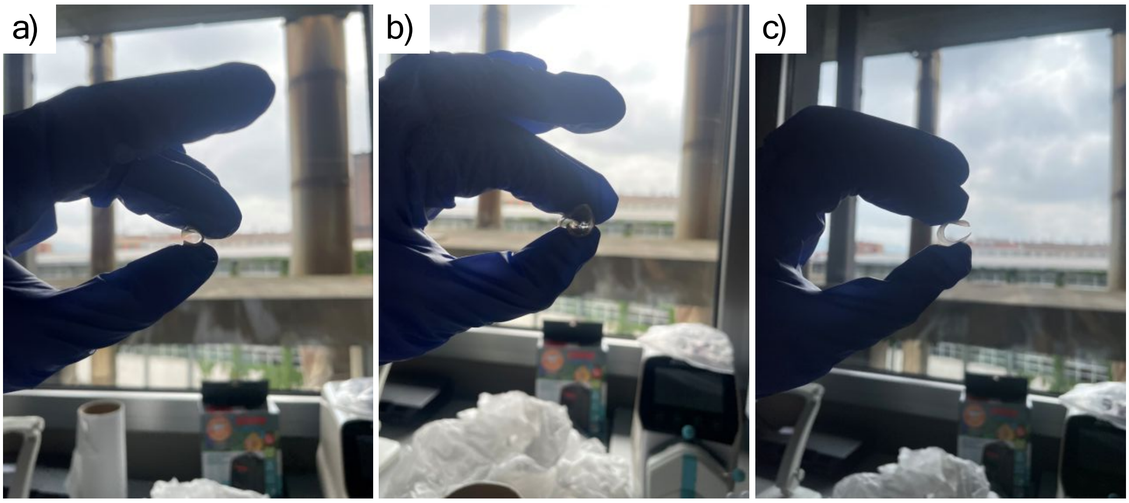

Figure S10. Optical photographs of the hydrogels showing the bendability of: a) HPC/CA; b) HPC/CA/25CNC; and c) HPC/CA/25ChNC.

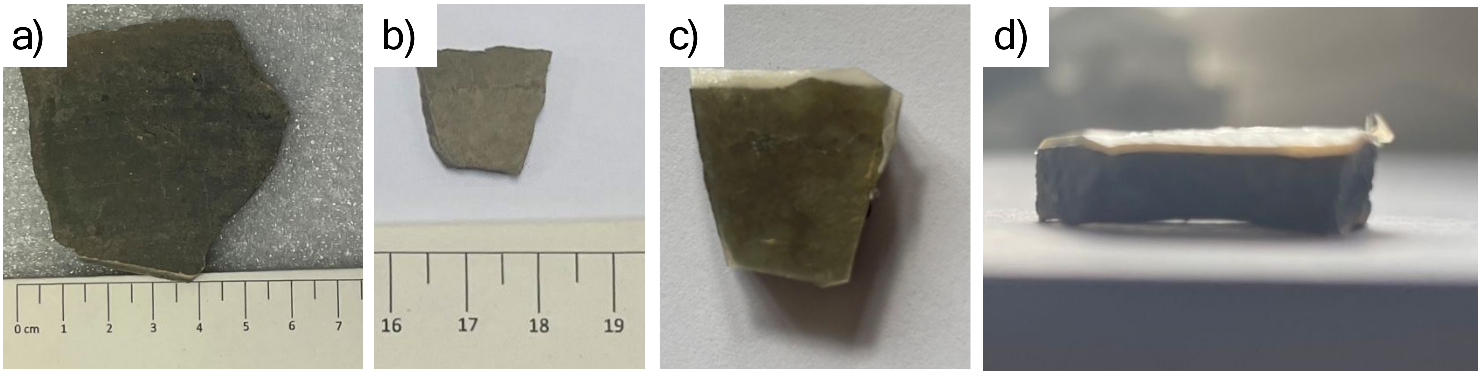

Figure S11. Optical photographs of cleaning application: a) optical photograph of the original "C ceramic"; b) "C ceramic" piece after cutting (~10 × 15 mm); c) top view; and d) side view.

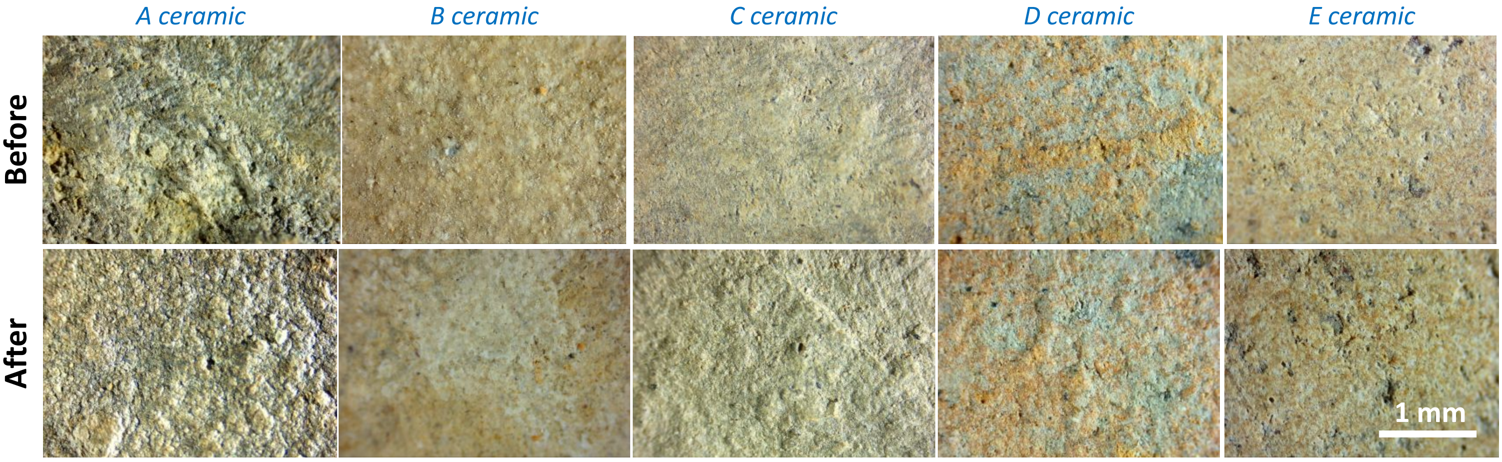

Figure S12. Optical microscope photographs (the scale bar is the same for all the images) showing the surface morphology of the five ceramics before and after application of the hydrogels. Note that the photographs were acquired at different locations on the ceramics.

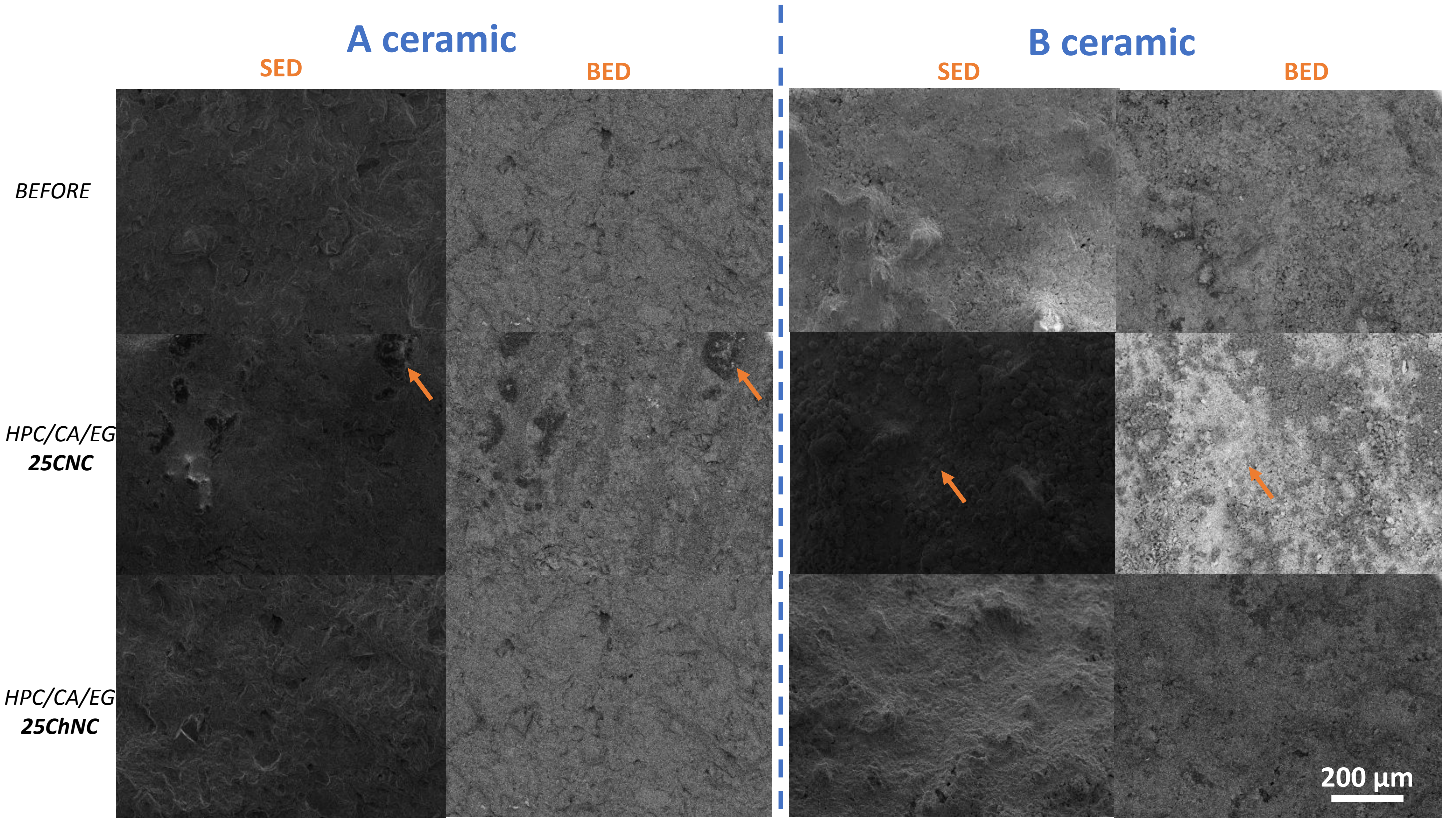

Figure S13. Representative FE-SEM micrographs using SED (secondary electron detector) and BED (backscattered electron detector) of: A and B ceramics for HPC/CA/EG/25CNC and HPC/CA/EG/25ChNC. Arrows indicate the presence of hydrogel residues.

Table S2. Quantitative WDXRF results shown in mass percentage. Iron content was expressed as total Fe_2O_3 amount. LOI indicates the mass loss that the ceramics suffer in the calcination process.

	Hydrogel	SiO_2	Al_2O_3	Fe_2O_3t	MnO	MgO	CaO	Na_2O	K_2O	TiO_2	P_2O_5	SO_3	PbO	LOI
	None	60.73	21.53	3.34	0.01	0.87	0.62	-	3.71	0.80	0.13	0.06	-	4.10
	HPC/CA	61.49	21.74	3.26	0.02	0.96	0.685	-	3.71	0.83	0.12	0.19	0.01	2.65
	HPC/CA/EG	61.59	21.91	3.18	0.01	0.88	0.62	-	3.76	0.83	0.13	0.12	-	2.45
A	HPC/CA/EG/10CNC	62.12	21.83	2.94	-	0.84	0.62	-	3.77	0.83	0.12	0.05	0.01	2.62
	HPC/CA/EG/25CNC	61.84	21.72	3.15	-	0.82	0.67	-	3.78	0.84	0.13	0.06	-	2.54
	HPC/CA/EG/10ChNC	63.06	22.19	2.97	-	0.82	0.65	-	3.82	0.86	0.13	0.06	-	2.11
	HPC/CA/EG/25ChNC	62.09	21.65	2.96	-	0.83	0.76	-	3.77	0.84	0.13	0.05	-	2.66
	None	53.56	11.07	3.66	0.05	1.72	12.98	0.29	1.30	0.53	0.18	0.14	0.39	9.65
	HPC/CA	53.91	10.99	3.48	0.02	1.02	14.87	0.27	1.65	0.54	0.18	0.08	0.27	9.41
	HPC/CA/EG	53.81	11.13	3.49	0.01	1.77	13.09	0.25	1.28	0.55	0.20	0.08	0.40	9.99
B	HPC/CA/EG/10CNC	54.26	11.25	3.54	0.01	1.26	14.09	0.30	1.33	0.55	0.17	0.11	0.34	9.10
	HPC/CA/EG/25CNC	55.58	11.57	3.59	0.01	1.42	14.69	0.29	1.30	0.57	0.13	0.11	0.11	9.00
	HPC/CA/EG/10ChNC	54.62	11.38	3.54	0.02	1.07	15.23	0.32	1.32	0.56	0.13	0.19	0.22	8.76
	HPC/CA/EG/25ChNC	55.50	11.43	3.60	0.01	1.06	15.04	0.29	1.29	0.57	0.09	0.15	0.23	8.10
	None	63.03	21.61	2.32	-	0.75	0.51	-	3.86	0.75	0.06	0.03	-	2.31
	HPC/CA	63.03	22.07	2.40	-	0.77	0.49	-	3.91	0.77	0.06	0.02	-	2.75
	HPC/CA/EG	62.84	21.68	2.32	-	0.75	0.47	-	3.86	0.75	0.06	0.03	-	2.42
C	HPC/CA/EG/10CNC	63.04	21.55	2.33	-	0.73	0.48	-	3.86	0.75	0.06	0.02	-	2.94
	HPC/CA/EG/25CNC	62.93	22.51	2.42	-	0.76	0.48	-	3.99	0.79	0.06	0.04	-	2.84
	HPC/CA/EG/10ChNC	62.10	22.45	2.46	-	0.74	0.48	-	4.04	0.80	0.06	0.03	-	2.41
	HPC/CA/EG/25ChNC	63.32	21.82	2.39	-	0.74	0.46	-	3.93	0.76	0.05	0.02	-	3.03
	None	66.81	12.21	4.81	0.04	0.62	5.18	0.27	2.28	0.68	0.24	0.05	0.77	3.16
	HPC/CA	64.51	11.72	4.66	0.03	0.56	5.20	0.27	2.11	0.66	0.29	0.08	3.27	3.45
	HPC/CA/EG	64.91	11.62	4.62	0.04	0.60	5.24	0.31	2.14	0.66	0.24	0.10	1.86	3.31
D	HPC/CA/EG/10CNC	64.92	11.76	4.65	0.03	0.56	5.34	0.29	2.14	0.68	0.26	0.07	1.80	3.35
	HPC/CA/EG/25CNC	66.43	11.98	4.74	0.03	0.62	5.07	0.32	2.18	0.70	0.25	0.08	2.15	3.13
	HPC/CA/EG/10ChNC	65.43	11.78	4.66	0.03	0.57	5.17	0.29	2.19	0.69	0.24	0.06	1.59	3.17
	HPC/CA/EG/25ChNC	65.02	11.56	4.65	0.04	0.58	5.21	0.28	2.19	0.67	0.25	0.09	1.84	3.40
	None	46.97	15.55	4.08	0.05	0.87	15.47	1.96	1.96	0.65	0.46	0.18	0.78	8.96
	HPC/CA	46.92	15.45	4.14	0.05	0.78	15.59	1.96	1.96	0.65	0.48	0.13	0.57	8.87
	HPC/CA/EG	46.18	15.08	4.02	0.05	0.78	15.55	1.92	1..92	0.63	0.32	0.26	0.75	9.38
E	HPC/CA/EG/10CNC	47.29	15.53	4.11	0.05	0.82	15.28	1.96	1.96	0.65	0.46	0.13	0.70	8.82
	HPC/CA/EG/25CNC	46.98	15.32	4.01	0.05	0.82	15.47	1.97	1.97	0.63	0.41	0.25	1.52	9.13
	HPC/CA/EG/10ChNC	46.23	14.90	3.98	0.05	0.76	15.33	1.93	1.93	0.63	0.46	0.15	1.77	9.05
	HPC/CA/EG/25ChNC	47.09	15.13	4.05	0.05	0.79	14.99	1.97	1.97	0.64	0.35	0.18	1.64	8.64

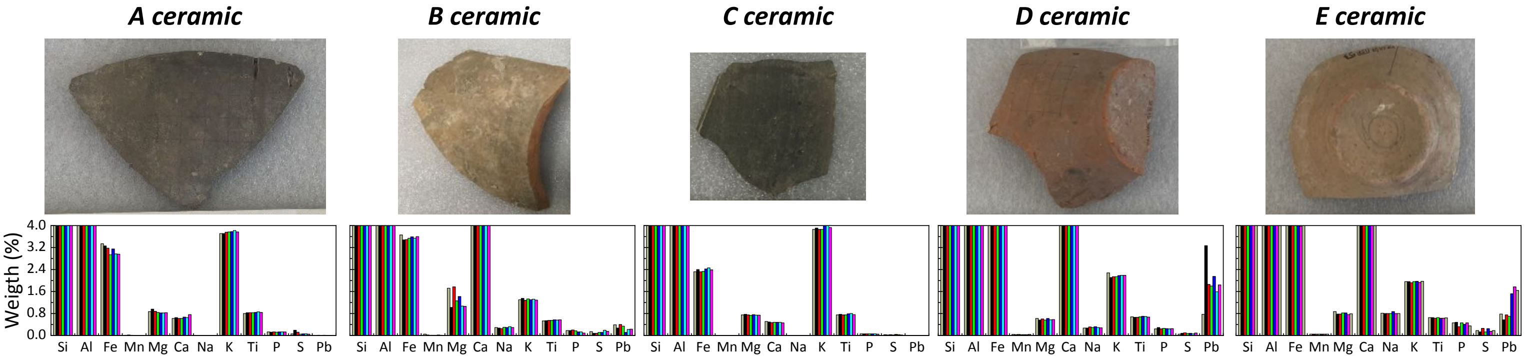

Figure S14. Magnified view of the semi-quantitative analysis of the uncleaned and cleaned ancient ceramics at the surface, as revealed by WDXRF. Note that the values shown for WDXRF are relative, so an increase in one element indicates a reduction in another.
